# Cu(II)-Catalyzed Homocouplings of (Hetero)Arylboronic Acids with the Assistance of 2-*O*-Methyl-d-Glucopyranose

**DOI:** 10.3390/molecules24203678

**Published:** 2019-10-12

**Authors:** Chunling Yuan, Li Zheng, Yingdai Zhao

**Affiliations:** Department of Medicinal Chemistry, Pharmacy School, Jinzhou Medical University, Jinzhou 121001, Liaoning, China; guihuaxiang856@sohu.com (L.Z.); ZYd1216221530@163.com (Y.Z.)

**Keywords:** 2-*O*-methyl-d-glucopyranose, copper catalyst, homocoupling, (hetero)arylboronic acids

## Abstract

This is the first report of a natural ligand improving the copper-catalyzed homocouplings of (hetero)arylboronic acids. Various important synthetic biaryl intermediates in organic synthesis could be assembled via this method. To gain insight into this reaction, in situ React IR technology was used to confirm the effectivity of this catalyst system. This protocol could provide important biaryl compounds in high yields within a short time.

## 1. Introduction

**Figure 1:**
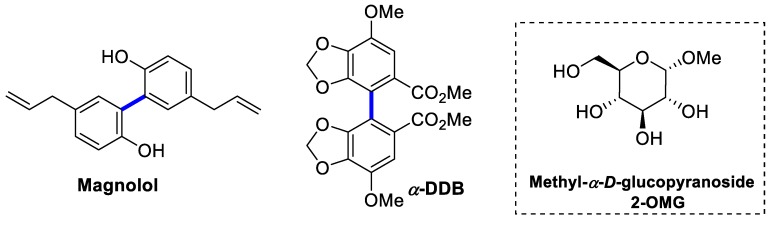
Chemical structures of some symmetrical biaryls and 2-OMG.

Symmetrical biaryls are ubiquitous reagents in organic synthesis and play a significant role in the pharmaceutical and chemical industries because of their unique bioactivities and chemical properties. As depicted in Figure 1, some natural symmetrical biaryl scaffolds have been discovered to display diverse biological activities, such as the antimicrobial agent *Honokiol* [1] and the hepatoprotective agent α-DDB [2]. The synthesis of symmetrical biaryl scaffolds mainly includes the use of metal-catalyzed homocoupling of aryl halides [3,4], boronic acids [5], aryl Grignard reagents [6], 1,2-diarylditellanes [7], and arenediazonium salts [8]. A common method for the preparation of symmetrical biaryls depends on palladium catalysis in the presence of related ligands, bases, and solvents [9]. Valiente et al. reported the aerobic homocoupling of arylboronic acids catalyzed by Pd(II) complexes, but this method requires special Pd(II)@MOF species, which limits its large-scale use [10]. Demir et al. found that copper(II) acetate could mediate the homocoupling of arylboronic acids [11]. Yamamoto and coworkers reported that 1,10-phenanthroline could accelerated the copper-catalyzed homocouplings of arylboronic acids, but this method suffered from low yields for some electron-withdrawing functionalities and substrates [12]. Singh et al. reported the ruthenium-catalyzed homocoupling of arylboronic acid in water [13]. Cu^II^–β-Cyclodextrin was discovered to catalyze the homocoupling and cross-coupling reactions of arylboronic acids [14], but the drawbacks of this method included a long reaction time and moderate yields.

2-*O*-Methyl-d-glucopyranose (2-OMG), a glucose analogue, is common in plants and contains multiple chirality centers; it was first reported as a promoter for copper catalyst. Herein we report on the development of conditions for the homocoupling of (hetero)arylboronic acids using a cheap and readily available copper catalyst with the assistance of 2-OMG. These reactions occurred at a mild temperature (40 °C), employing substituted arylboronic acids and inexpensive Cu(II) acetate, with high yields. This wide scope allows for the preparation of many symmetrical biaryls. To our delight, some substituted arylboronic acids also have good tolerance and moderate to high yields, and some coupling results could be preliminarily summarized that were advantageous for the preparation of symmetrical biaryls.

## 2. Results and Discussion

Our investigations began with the homocoupling of phenylboronic acid in the presence of copper salts, using 2-OMG as a ligand (Table 1). A screen of copper salts revealed that no biphenyl was observed when the reaction was conducted at 40 °C for 1 h with CuI, CuO, or powdered Cu as the catalyst (entries 1–3). However, CuSO_4_ slightly promoted the homocoupling reaction, and a lower yield of homocoupling products was observed 10% (entry 4). Replacing CuSO_4_ with Cu(OAc)_2_ offered an opportunity to implement a synthesis of symmetrical biaryls such as biphenyl directly from phenylboronic acid. Attempts to optimize the reaction through modification of the base proved unsuccessful, and we did not find any effects of bases on the reaction in terms of yield (entries 5–7). Practically, the coupling reaction could process smoothly without any assistance of bases, yielding biphenyl at 99% (entry 8). Different solvents including DMF, dioxane, H_2_O, and methanol were tested, with DMF being the most effective in the screening reaction (entries 9–11). Notably, the use of H_2_O was detrimental, as an unexpected byproduct phenol was obtained. Lowering the temperature to room temperature prolonged the reaction time without impacting the yield (entry 12). Control experiments (entry 13) confirmed that 2-OMG could indeed promote the Cu(OAc)_2_-catalyzed coupling reaction, forming biphenyl at a high yield within a short time at room temperature. However, under basic conditions in the absence of 2-OMG, the desired product was obtained at a 58% yield. The loading catalyst investigation showed that the 5% loading catalyst needed longer reaction time, but did not impact the yield compared to a 10% loading catalyst (entry 14). Further increasing it to a 15% loading did not significantly accelerate the reaction (entry 15). The scalability of this protocol was tested through the synthesis of biphenyl **2a** on a 10-fold scale. In this case, the catalyst loading was lowered to 5 mol % and the desired biphenyl was obtained at 98% isolated yield (entry 16).

**Figure 2:**
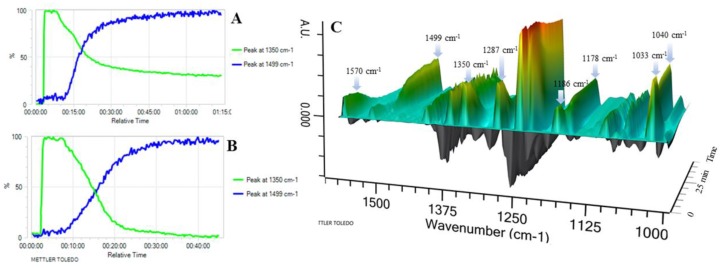
2D trends and 3D surface of ReactIR experiment. (**A**) Reaction conducted without using 2-OMG; (**B**) reaction conducted with the use of 2-OMG. Y axis indicates the intensity of the signal; (**C**) 3D surface figure was obtained with the use of 2-OMG.

In situ React IR technology was employed to monitor the conversion of 4-methoxyphenylboronic acid (**1f**) to the corresponding 4,4′-dimethoxy-1,1′-bipheny (**2f**). As seen in Figure 2 (A and B: 2D trends; C: 3D surface), less than 5 min after the addition of 2-OMG and Cu(OAc)_2_ to the 4-methoxyphenylboronic acid start heating, some new, sharp peaks appeared at 1040, 1178, and 1499 cm^−1^, indicating the appearance of the product. The intensity of these peaks gradually increased as the reaction temperature was raised to 40 °C. Importantly, the disappearance of the 1033, 1186, 1287, 1350, and 1570 cm^−1^ bands was marked by the appearance of three bands at 1723, 1622, and 1611 cm^−1^, corresponding to 4,4′-dimethoxy-1,1′-bipheny (Figure 2C). The conversion of 4-methoxyphenylboronic acid reached 50% after only 15 min, and 100% after 40 min (Figure 2B). In contrast, a control experiment (without 2-OMG) showed that the conversion of 4-methoxyphenylboronic acid reached only 65% after 75 min; complete conversion required higher temperatures and a longer time (Figure 2A). Thus, it was concluded that the conversion of 4-methoxyphenylboronic acid to the corresponding 4,4′-dimethoxy-1,1′-bipheny underwent quick coupling to produce the target compound.

**Scheme 1:**
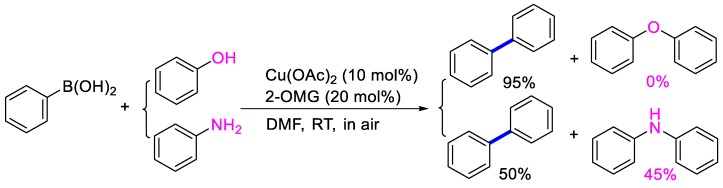
Attempted cross-coupling of phenol or aniline with phenylboronic acid.

In the optimized conditions, the scope of the (hetero)arylboronic acids was investigated (Table 2). A range of functional groups on the substituted (hetero)arylboronic acids proved to be compatible, including phenyl, methyl, methoxy, hydroxyl, and ester (**1b**−**1i**, **1p**). The pyridine boronic acid and substituted pyridine boronic acid could also be applied in the reaction (**1m**−**1o**). Substrates bearing either electron-withdrawing or electron-donating groups on the aromatic ring cross-coupled with universally high yields; however, substrates bearing electron-donating groups generally required a longer reaction time. The introduction of a second methoxy group at the aromatic ring was also tolerated, while the reaction stopped at 80% conversion, presumably due to the strong electron-donating effect of **1g**. The presence of an *ortho* substituent on the aromatic ring decreased the yield of the homocoupling product (**1d**, **1h**). Fluorine substituents were well tolerated, and excellent results were observed (**1j**–**1l**, **1n**–**1o**). The cross-coupling reactions of phenylboronic acids with phenols for the synthesis of biaryl ethers failed to react (Scheme 1), proving that the hydroxyl group was broadly tolerant to the homocoupling (**1h**, **1i**). However, a limitation of the reaction was that amino-substituted (hetero)arylboronic acids led to a complex reaction due to the production of biaryls and diphenylamines (Scheme 1).

**Scheme 2:**
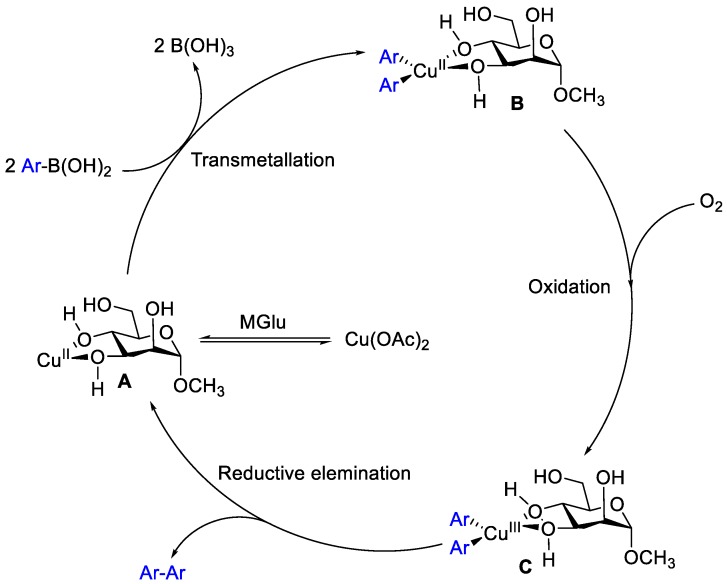
Plausible mechanism for the homocoupling of arylboronic acid.

The mechanism of the homocoupling was analogous to the catalytic cycle with previous report [11,15], a plausible reaction pathway was proposed as shown in Scheme 2. The reaction was initiated by the coordination of the oxygen atoms from MGlu to the copper center, forming a catalytically active complex. Then, double transmetallation of Cu^II^ with two molecules of (hetero)arylboronic acids afforded Ar–Cu(II)–Ar, which undergoes oxidation by air to yield a Cu^III^ intermediate, the reductive elimination of which released the homocoupled product Ar–Ar.

## 3. Materials and Methods

All of the starting materials, reagents, and solvents are commercially available and were used without further purification. Melting points were determined with an X-4 apparatus and were uncorrected (Beijing Taike Instrument Co., Ltd, Beijing, China). The nuclear magnetic resonance (NMR) spectra were recorded on a Bruker 400 MHz spectrometer (Bruker Technology Co., Ltd., Karlsruhe, Germany) in CDCl_3_ or DMSO-*d_6_* using tetramethylsilane (TMS) as an internal standard. Electrospray ionization mass spectrometry (MS (ESI)) analyses were recorded in an Agilent 1100 Series MSD Trap SL (Santa Clara, CA, USA). The reactions were monitored by thin-layer chromatography (TLC: HG/T2354-92, GF254), and compounds were visualized with UV light.

### 3.1. React IR Experiment 

React IR experiments were conducted using a Mettler Toledo ReactIR 15 with MCT detector (Mettler Toledo International Trading Co., Ltd., Zurich, Switzerland) using HappGenzel; DiComp (Diamond) probe connected via AgX × 9.5 mm × 1.5 m fiber (silver halide). iC IR 4.3 reaction analysis software (Mettler Toledo International Trading Co., Ltd., Zurich, Switzerland.) was used during data collection and analysis.

To a 50 mL, three-neck flask equipped with a magnetic stirrer was inserted the ReactIR 15 DiComp probe. IR spectra were obtained every 15 s. Data collection began at the start of the experiment. To a solution of 4-methoxyphenylboronic acid (0.40 g, 2.63 mmol) in DMF (5 mL) and at room temperature was added 2-OMG (102 mg, 0.53 mmol) and Cu(OAc)_2_ (48 mg, 0.26 mmol). The resulting solution was gradually heated to 40 °C and stirred for 60 min. The workup was consistent with the general procedure for catalytic experiments.

### 3.2. General Procedure for Catalytic Experiments

To a solution of (hetero)arylboronic acids (2.63 mmol), and 2-OMG (20 mol %) in DMF (5 mL) was added Cu(OAc)_2_ (10 mol %) in air. The mixture was heated to 40 °C with stirring for the indicated time. The reactor was cooled to room temperature, and the reaction mixture was poured into water (20 mL), extracted with ethyl acetate (20 mL × 3), and the organic layer washed with water (20 mL × 2) and once with brine (25 mL), dried over magnesium sulfate, and concentrated in vacuo. The product was purified by flash column chromatography on silica gel using petroleum ether as the eluent.

*Biphenyl* (**2a**) [16]: white solid (397 mg, 98%), m.p. 69–70 °C. ^1^H NMR (400 MHz, CDCl_3_) δ: 7.60 (t, *J* = 1.2 Hz, 2H), 7.44 (t, *J* = 7.2 Hz, 2H), 7.34 (t, *J* = 7.4 Hz, 1H). ^13^C NMR (150 MHz, CDCl_3_) δ: 141.2, 128.7, 127.2, 127.1.

*4-Quaterphenyl* (**2b**) [17]: white solid (780 mg, 97%), m.p. > 300 °C. ^1^H NMR (400 MHz, CDCl_3_) δ: 7.77–7.37 (m, 18H). ^13^C NMR (150 MHz, CDCl_3_) δ: 140.7, 140.3, 139.6, 128.8, 127.6, 127.5, 127.1.

*4,4′-Dimethyl-1,1′-biphenyl* (**2c**) [18]: white solid (450 mg, 94%), m.p. 121–123 °C. ^1^H NMR (600 MHz, CDCl_3_) δ: 7.45 (d, *J* = 7.7 Hz, 2H), 7.24 (d, *J* = 8.2 Hz, 2H), 2.38 (s, 6H). ^13^C NMR (150 MHz, CDCl_3_) δ: 138.3, 136.7, 129.4, 126.8, 21.1.

*2,2′-Dimethoxy-1,1′-biphenyl* (**2d**) [18]: white solid (473 mg, 84%). m.p. 178–179 °C. ^1^H NMR (400 MHz, CDCl_3_) δ: 7.35–7.31 (m, 2H), 7.26–7.02 (m, 2H), 7.00 (q, *J* = 7.4, 6.6 Hz, 4H), 3.77 (s, 6H). ^13^C NMR (150 MHz, CDCl_3_) δ: 157.0, 131.4, 128.6, 127.8, 120.3, 111.1, 55.7.

*3,3′-Dimethoxy-1,1′-biphenyl* (**2e**) [18]: colorless liquid (507 mg, 90%). ^1^H NMR (600 MHz, CDCl_3_) δ: 7.32 (t, *J* = 7.9 Hz, 2H), 7.17–7.15 (m, 2H), 7.11 (t, *J* = 2.2 Hz, 2H), 6.88–6.86 (m, 2H), 3.81 (s, 6H). ^13^C NMR (150 MHz, CDCl_3_) δ: 159.9, 142.6, 129.7, 119.7, 112.9, 112.8, 55.2.

*4,4′-Dimethoxy-1,1′-biphenyl* (**2f**) [18]: white solid (512 mg, 91%), m.p. 177–179 °C. ^1^H NMR (400 MHz, CDCl_3_) δ: 7.49–7.45 (m, 4H), 6.97–6.93 (m, 4H), 3.84 (s, 6H). ^13^C NMR (100 MHz, CDCl_3_) δ: 158.7, 133.5, 127.7, 114.2, 55.4.

*3,3′,5,5′-Tetramethoxy-1,1’-biphenyl* (**2g**) [11]: colorless liquid (641 mg, 89%). ^1^H NMR (400 MHz, CDCl_3_) δ: 7.18 (t, *J* = 8.3 Hz, 2H), 6.52 (d, *J* = 2.4 Hz, 2H), 6.50 (d, *J* = 2.4 Hz, 2H), 6.47 (t, *J* = 2.3 Hz, 2H), 3.79 (s, 12H). ^13^C NMR (100 MHz, CDCl_3_) δ: 160.9, 129.9, 106.2, 100.5, 55.3.

*[1,1′-Biphenyl]-2,2′-diol* (**2h**) [19]: white solid (445 mg, 91%), m.p. 108–109 °C. ^1^H NMR (400 MHz, CDCl_3_) δ: 7.38–7.33 (m, 2H), 7.30 (d, *J* = 1.4 Hz, 1H), 7.10–7.05 (q, *J* = 8.6, 1.0 Hz, 4H), 5.40 (br, 2H). ^13^C NMR (150 MHz, CDCl_3_) δ: 152.0, 130.6, 129.2, 122.9, 120.9, 115.9.

*[1,1′-Biphenyl]-4,4′-diol* (**2i**) [20]: white solid (455 mg, 93%), m.p. 229–230 °C. ^1^H NMR (400 MHz, DMSO*-d_6_*) δ: 9.38 (s, 2H), 7.37 (d, *J* = 8.5 Hz, 4H), 6.79 (d, *J* = 8.5 Hz, 4H). ^13^C NMR (150 MHz, DMSO-*d_6_*) δ: 155.7, 130.5, 126.3, 115.0.

*4,4′-Difluoro-1,1′-biphenyl* (**2j**) [18]: white solid (490 mg, 98%), m.p. 86–88 °C. ^1^H NMR (400 MHz, CDCl_3_) δ: 7.43–7.38 (m, 2H), 7.36–7.33 (m, 2H), 7.28–7.27 (m, 1H), 7.26–7.25 (m, 1H), 7.09–7.04 (m, 2H). ^13^C NMR (100 MHz, CDCl_3_) δ: 163.2 (*J* = 244.5 Hz), 142.2 (*J* = 7.6 Hz), 130.4 (*J* = 8.4 Hz), 122.7 (*J* = 2.8 Hz), 114.8, 114.6, 114.2, 113.9.

*3,3′-Difluoro-1,1′-biphenyl* (**2k**) [18]: colorless liquid (490, 98%). ^1^H NMR (400 MHz, CDCl_3_) δ: 7.50–7.47 (m, 4H), 7.14–7.09 (m, 2H). ^13^C NMR (100 MHz, CDCl_3_) δ: 162.4 (*J* = 244.9 Hz), 136.4 (*J* = 3.1 Hz), 128.6 (*J* = 8.4 Hz), 115.6 (*J* = 21.3 Hz).

*3,3′-Bis(trifluoromethyl)-1,1′-biphenyl* (**2l**) [21]: white solid (755 mg, 99%). ^1^H NMR (400 MHz, CDCl_3_) δ: 7.83 (s, 2H), 7.77 (d, *J* = 7.6 Hz, 2H), 7.66 (d, *J* = 7.8 Hz, 2H), 7.60 (t, *J* = 7.7 Hz, 2H). ^13^C NMR (150 MHz, CDCl_3_) δ: 140.6, 131.3 (*J* = 64.3 Hz), 130.5, 129.5, 124.7 (*J* = 7.5 Hz), 124.0 (*J* = 270.0 Hz), 123.9 (*J* = 7.6 Hz).

*2,2′-Bipyridine* (**2m**) [18]: white solid (386 mg, 94%), m.p. 71 - 72 °C. ^1^H NMR (600 MHz, CDCl_3_) δ: 8.71 (d, *J* = 4.3 Hz, 2H), 8.44 (d, *J* = 7.9 Hz, 2H), 7.85 (t, *J* = 7.6 Hz, 2H), 7.34 (t, *J* = 5.4 Hz, 2H). ^13^C NMR (150 MHz, CDCl_3_) δ: 156.0, 149.1, 137.1, 123.8, 121.2.

*6,6′-Difluoro-3,3′-bipyridine* (**2n**): white solid (480 mg, 95%), m.p. 106–108 °C. ^1^H NMR (400 MHz, CDCl_3_) δ: 8.40 (d, *J* = 1.7 Hz, 2H), 7.98–7.93 (m, 2H), 7.07 (dd, *J* = 8.4, 3.0 Hz, 2H). ^13^C NMR (100 MHz, CDCl_3_) δ: 163.6 (*J* = 239.6 Hz), 145.9 (*J* = 15.1 Hz), 139.7 (*J* = 8.1 Hz), 130.6 (*J* = 4.6 Hz), 110.2 (*J* = 4.6 Hz), 110.2, 109.8.

*5,5′-Difluoro-2,2′-bipyridine* (**2o**) [22]: white solid (470 mg, 93%), m.p. 151–152 °C. ^1^H NMR (600 MHz, CDCl_3_) δ: 8.50 (d, *J* = 2.3 Hz, 2H), 8.39 (dd, *J* = 8.8, 4.4 Hz, 2H), 7.54–7.51 (m, 2H). ^13^C NMR (150 MHz, CDCl_3_) δ: 160.7, 159.0, 151.4, 151.42, 137.3, 137.1, 123.9, 123.8, 122.3, 122.2.

*Dimethyl [1,1′-biphenyl]-4,4′-dicarboxylate* (**2p**) [23]: white solid (639 mg, 90%), m.p. 211–213 °C. ^1^H NMR (400 MHz, CDCl_3_) δ: 8.15 (d, *J* = 8.4 Hz, 4H), 7.72 (q, *J* = 8.4, 1.6Hz, 4H), 3.97 (s, 6H). ^13^C NMR (150 MHz, DMSO-*d_6_*) δ: 167.4, 143.7, 130.4, 129.1, 127.8, 52.7.

Copies of ^1^H NMR and ^13^C NMR are available in supplementary materials.

## 4. Conclusions

In summary, we have developed a catalyst system for the homocoupling of (hetero)arylboronic acids that utilizes readily available (hetero)arylboronic acids and inexpensive Cu(OAc)_2_ in the presence of 2-OMG, with a moderate to high yield. The natural ligand could indeed promote copper-catalyzed homocouplings through an in situ IR reactor. The naturally available ligand (2-OMG) contains chiral centers, which could pave the way for the enantioselective synthesis of many important optically active molecules. Efforts to understand the crucial factors responsible for the improved reactivity and to expand the scope of the homocoupling of (hetero)arylboronic acids to other classes of aromatic compounds are currently underway.

## Figures and Tables

**Table 1:**
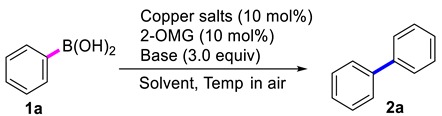
Identification of the reaction conditions ^a.^

Entry	Catalyst (mol %)	Base	Solvent	Temp (°C)	Yield (%) ^b^
1	CuI (10)	K_2_CO_3_	DMF	40	-- ^c^
2	CuO (10)	K_2_CO_3_	DMF	40	-- ^c^
3	Powdered Cu (10)	K_2_CO_3_	DMF	40	-- ^c^
4	CuSO_4_ (10)	K_2_CO_3_	DMF	40	10
5	Cu(OAc)_2_ (10)	K_2_CO_3_	DMF	40	85
6	Cu(OAc)_2_ (10)	Cs_2_CO_3_	DMF	40	80
7	Cu(OAc)_2_ (10)	Et_3_N	DMF	40	78
8	Cu(OAc)_2_ (10)	--	DMF	40	99
9	Cu(OAc)_2_ (10)	--	Dioxane	40	-- ^c^
10	Cu(OAc)_2_ (10)	--	H_2_O	40	-- ^c^
11	Cu(OAc)_2_ (10)	--	MeOH	40	14
12	Cu(OAc)_2_ (10)	--	DMF	RT ^d^	98
13 ^e^	Cu(OAc)_2_ (10)	--	DMF	40	58
14 ^f^	Cu(OAc)_2_ (5)	--	DMF	40	96
15	Cu(OAc)_2_ (15)	--	DMF	40	98
16 ^g^	Cu(OAc)_2_ (5)	--	DMF	RT ^d^	98

^a^ Reactions conducted on phenylboronic acid (2.63 mmol), catalyst (10 mol %), 2-OMG (10 mol %), solvent (5 mL), base (7.89 mmol), for 1 h, unless otherwise noted. ^b^ Isolated yield with column chromatography. ^c^ Almost no reactions were observed by TLC. ^d^ RT = room temperature. ^e^ Without 2-OMG. ^f^ Reaction time was 24 h. ^g^ The loading of phenylboronic acid was 26.3 mmol, the catalyst loading was 5 mol %, and the reaction time was 24 h.

**Table 2:**
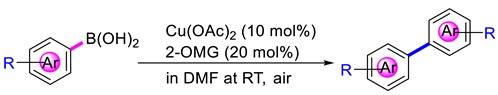
Scope with respect to (hetero)arylboronic acids ^a^.

Entry	Substance	Product	Time (min)	Yield (%) ^b^
1	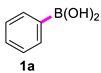	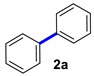	60	98
2	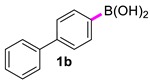	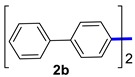	60	97
3	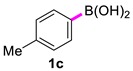	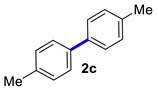	60	94
4	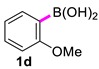	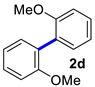	90	84
5	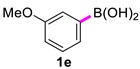	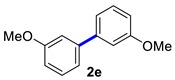	90	90
6	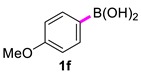	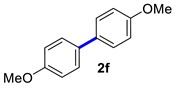	60	91
7	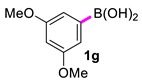	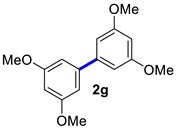	90	89
8	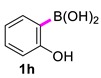	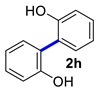	90	91
9	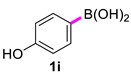	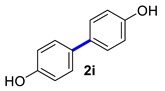	90	93
10	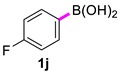	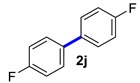	60	98
11	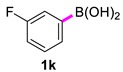	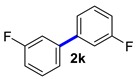	60	98
12	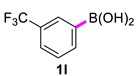	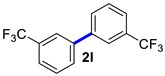	60	99
13	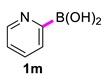	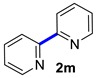	90	94
14	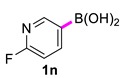	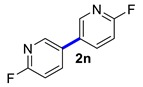	90	95
15	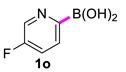	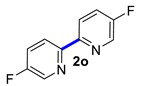	90	93
16	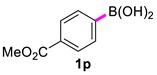	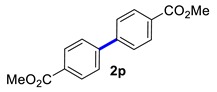	90	90

^a^ Reactions conducted on (hetero)arylboronic acids (2.63 mmol), Cu(OAc)_2_ (10 mol %), 2-OMG (20 mol %), DMF (5 mL) in air, unless otherwise noted. ^b^ Isolated yield with column chromatography.

## Supplementary Materials

The following are available online. Copies of ^1^H NMR and ^13^C NMR for all compounds were placed in supporting information.
