# Supplementary material for: Cu(II)-Catalyzed Homocouplings of (Hetero)Arylboronic Acids with the Assistance of 2-O-Methyl-d-Glucopyranose
Source: Molecules. 2019 Oct 12;24(20):3678. doi: 10.3390/molecules24203678 (PMC6832226; doi:10.3390/molecules24203678)
Supplement: Supplementary file 1 [file molecules-24-03678-s001.pdf]

Supporting information

Cu(II)-Catalyzed Homocouplings of (Hetero)Arylboronic Acids with the assistant of 2-*O*-Methyl-D-glucopyranose

Chunling Yuan *, Li Zheng and Yingdai Zhao

Department of Medicinal Chemistry, Pharmacy School, Jinzhou Medical University, Jinzhou 121001, Liaoning, PR China

*Correspondence: yuanchunling@jzmu.edu.cn; Tel.: +86-0416-4673440; +86-13898354237

Copies of ^{1}H NMR and ^{13}C NMR

Compound 2a 1
Compound 2b 2
Compound 2c 3
Compound 2d 4
Compound 2e 5
Compound 2f 6
Compound 2g 7
Compound 2h 8
Compound 2i 9
Compound 2j 10
Compound 2k 11
Compound 2l 12
Compound 2m 13
Compound 2n 14
Compound 2o 15
Compound 2p 16

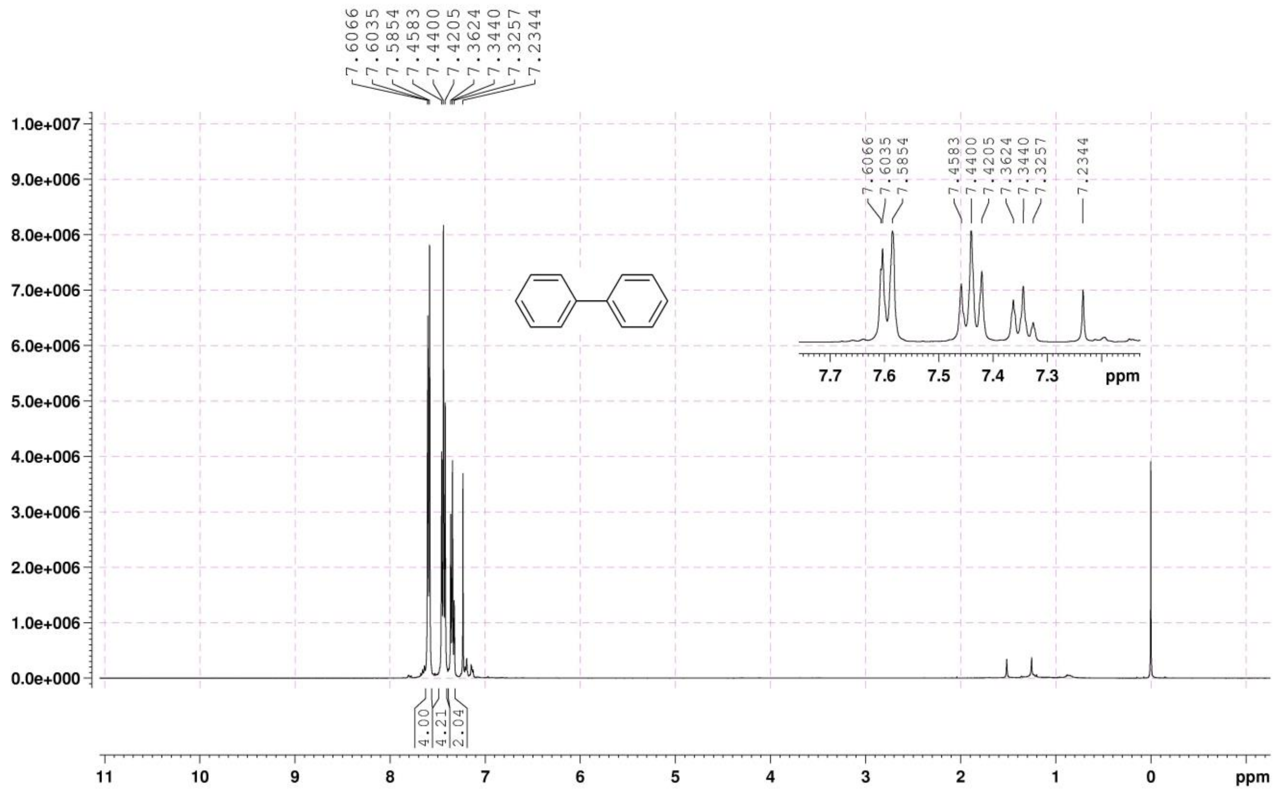
7.6066
7.6035
7.5854
7.4583
7.4400
7.4205
7.3624
7.3440
7.3257
7.2344
4.00
4.21
2.04
ppm

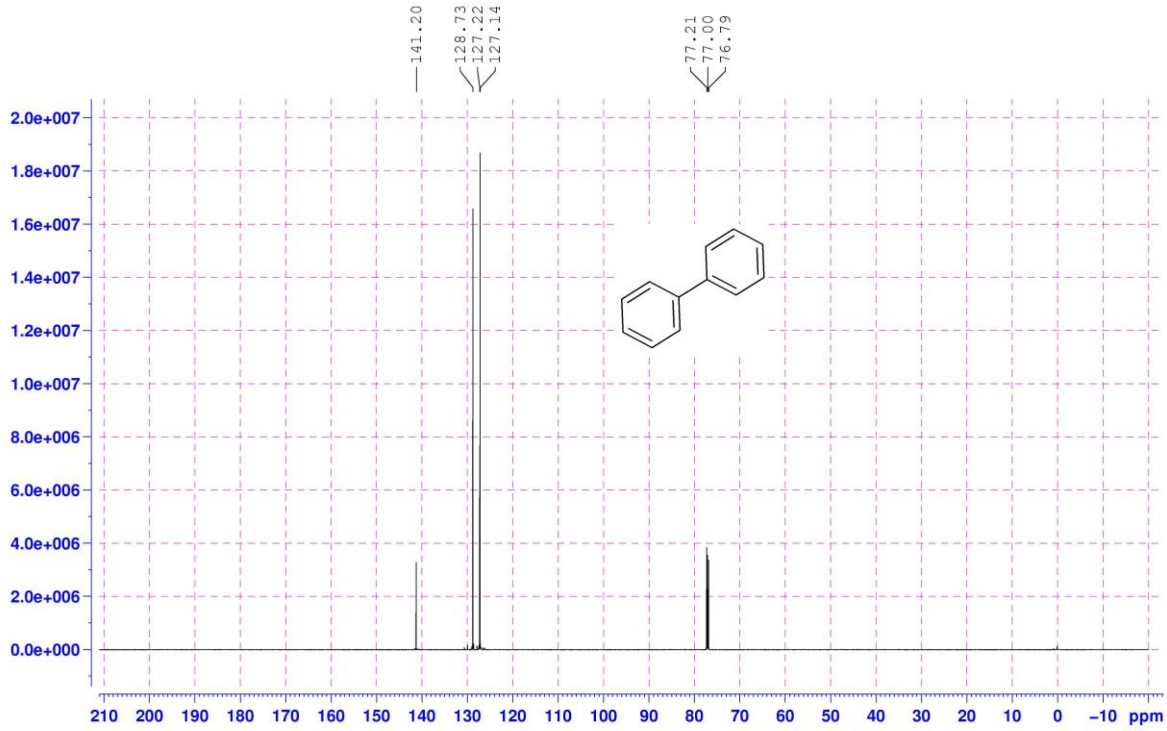
141.20
128.73
127.22
127.14
77.21
77.00
76.79
ppm

Compound 2a

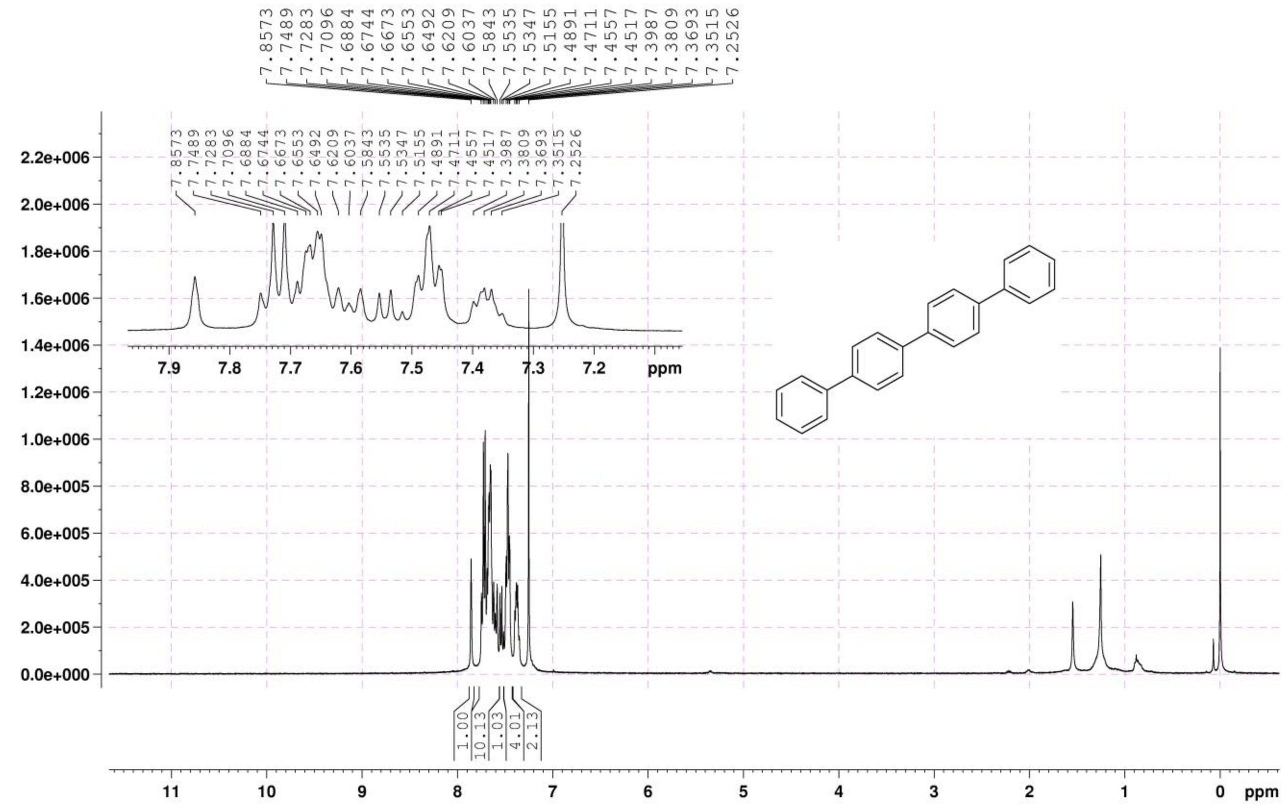
7.8573
7.7489
7.7283
7.7096
7.6884
7.6744
7.6673
7.6553
7.6492
7.6209
7.6037
7.5843
7.5535
7.5347
7.5155
7.4891
7.4711
7.4557
7.4517
7.3987
7.3809
7.3693
7.3515
7.2526
1.00
10.13
1.03
4.01
2.13
ppm

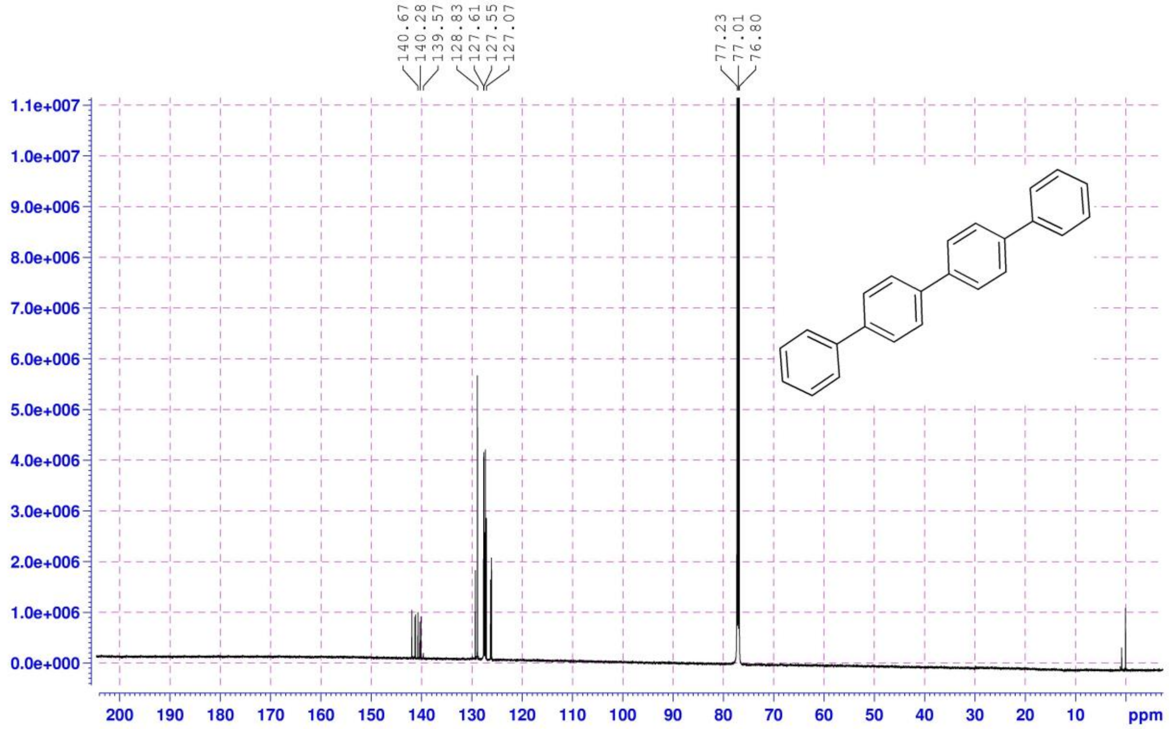
140.67
140.28
139.57
128.83
127.61
127.55
127.07
77.23
77.01
76.80
ppm

Compound 2b

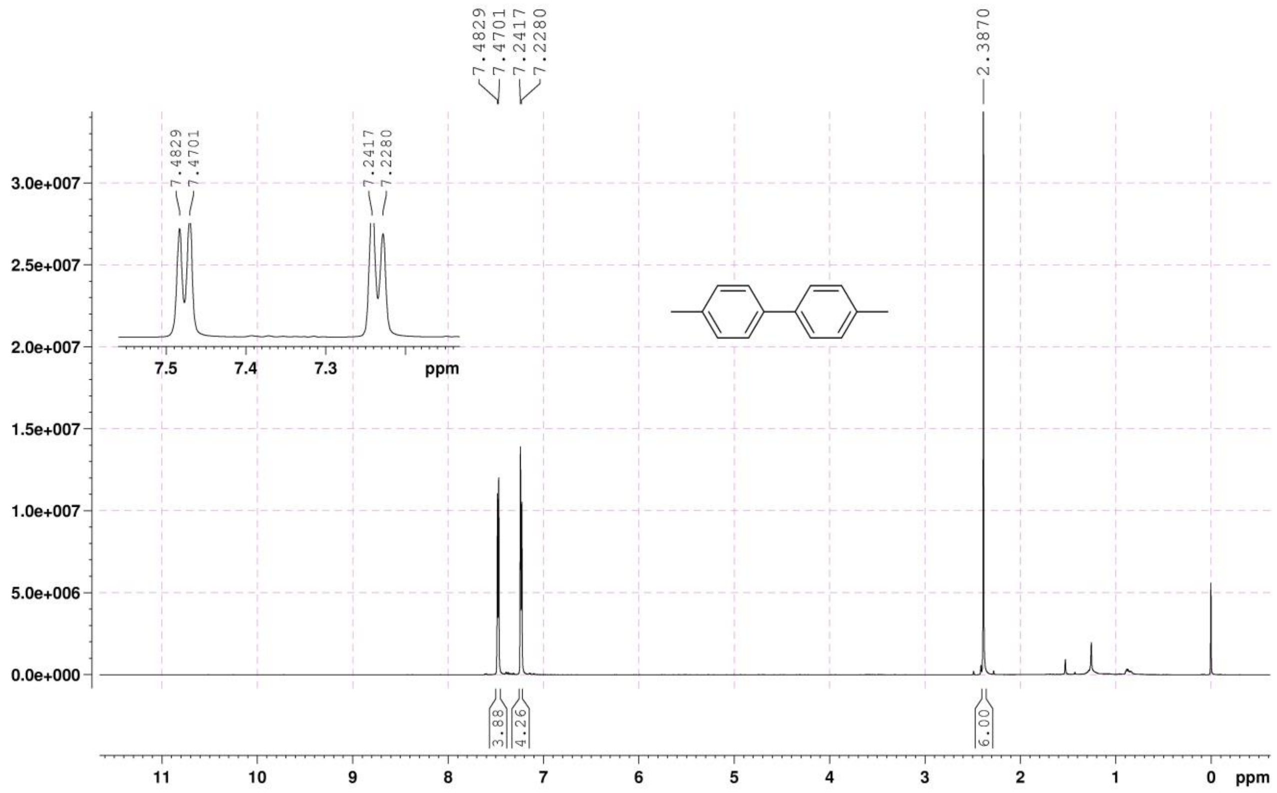

7.4829
7.4701
7.2417
7.2280
2.3870
3.0e+007
2.5e+007
2.0e+007
1.5e+007
1.0e+007
5.0e+006
0.0e+000
7.5
7.4
7.3
ppm
3.88
4.26
6.00
11
10
9
8
7
6
5
4
3
2
1
0
ppm

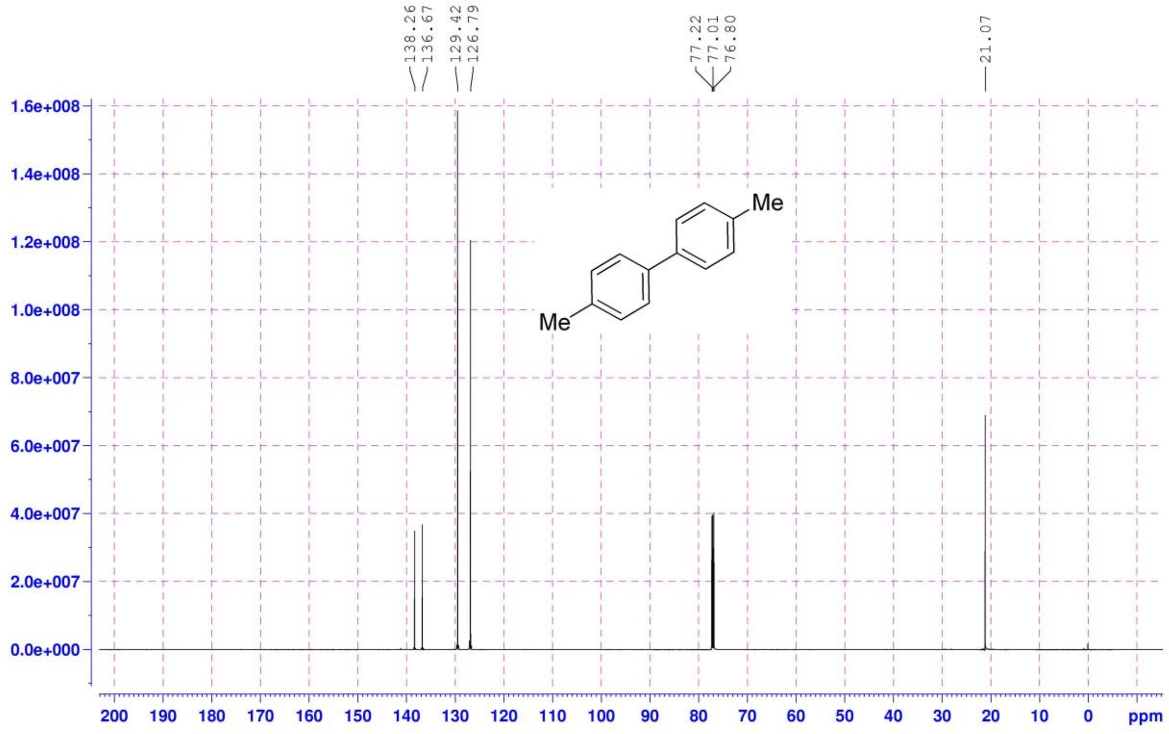

138.26
136.67
129.42
126.79
77.22
77.01
76.80
21.07
1.6e+008
1.4e+008
1.2e+008
1.0e+008
8.0e+007
6.0e+007
4.0e+007
2.0e+007
0.0e+000
Me
Me
200
190
180
170
160
150
140
130
120
110
100
90
80
70
60
50
40
30
20
10
0
ppm

Compound 2c

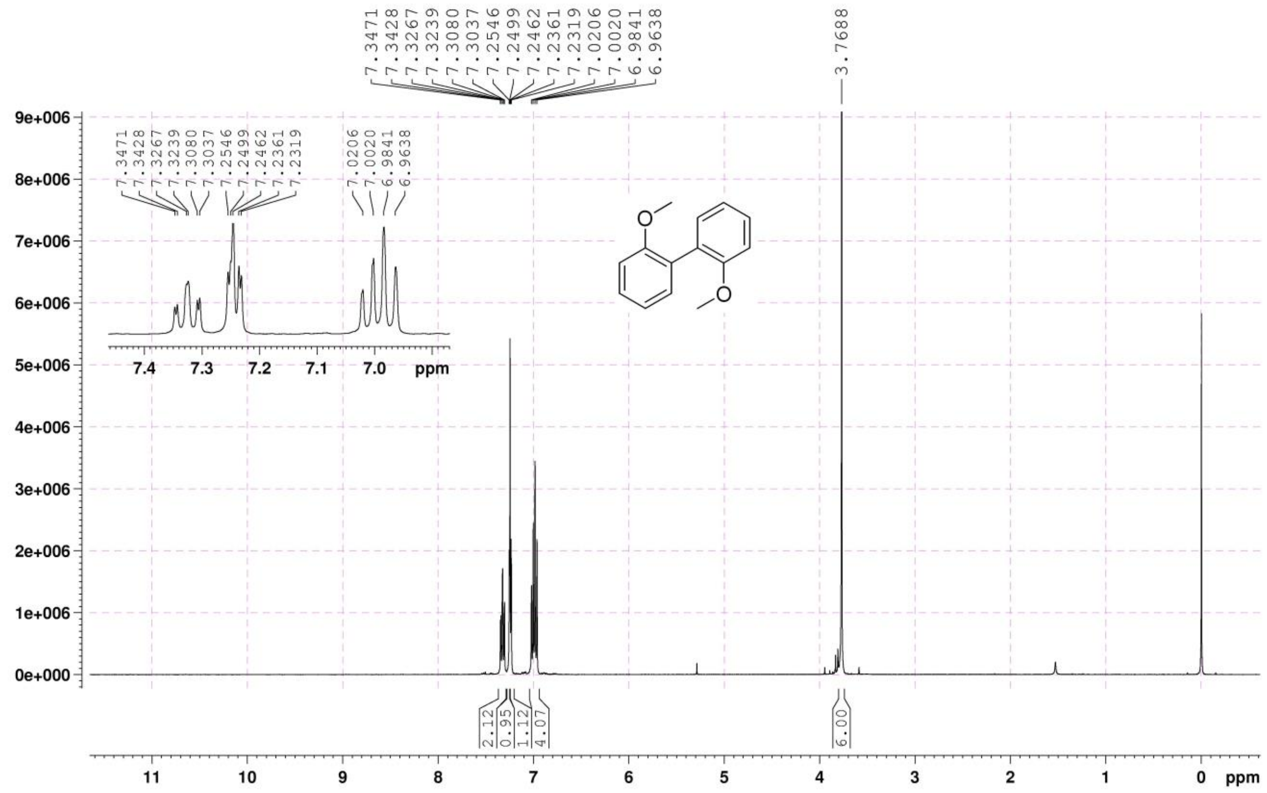
7.3471
7.3428
7.3267
7.3239
7.3080
7.3037
7.2546
7.2499
7.2462
7.2361
7.2319
7.0206
7.0020
6.9841
6.9638
3.7688
2.12
0.95
1.12
4.07
6.00
ppm

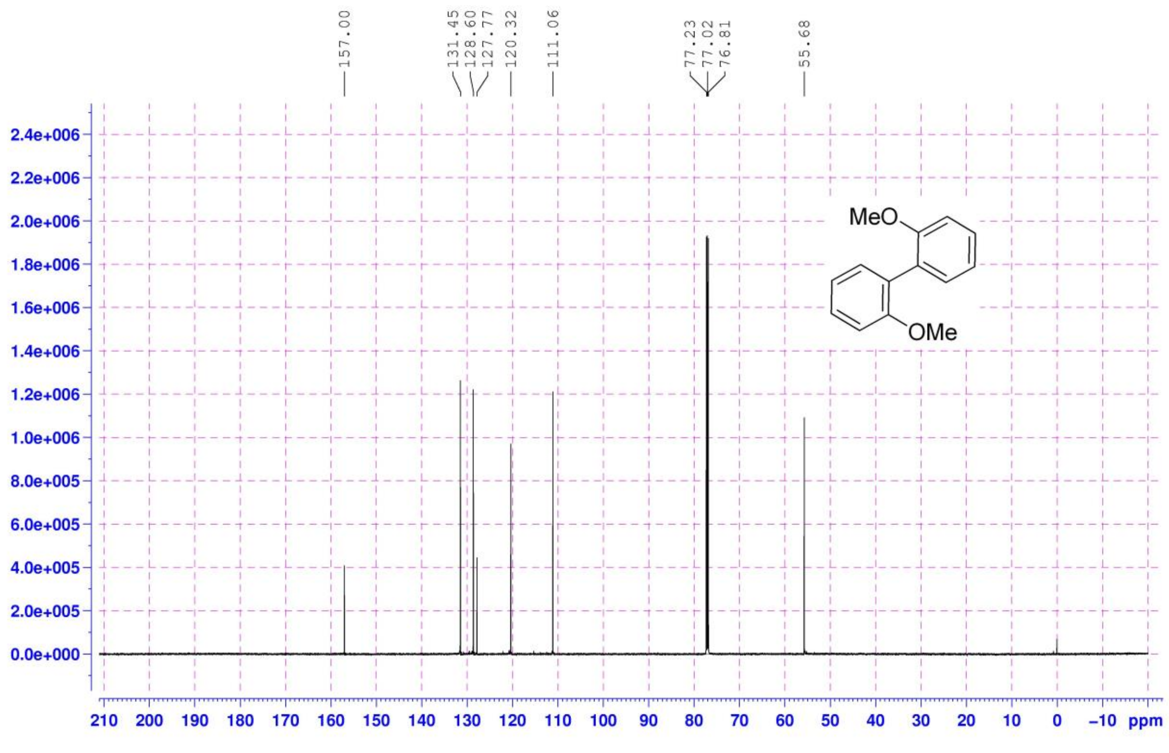
157.00
131.45
128.60
127.77
120.32
111.06
77.23
77.02
76.81
55.68
MeO
OMe
ppm

Compound 2d

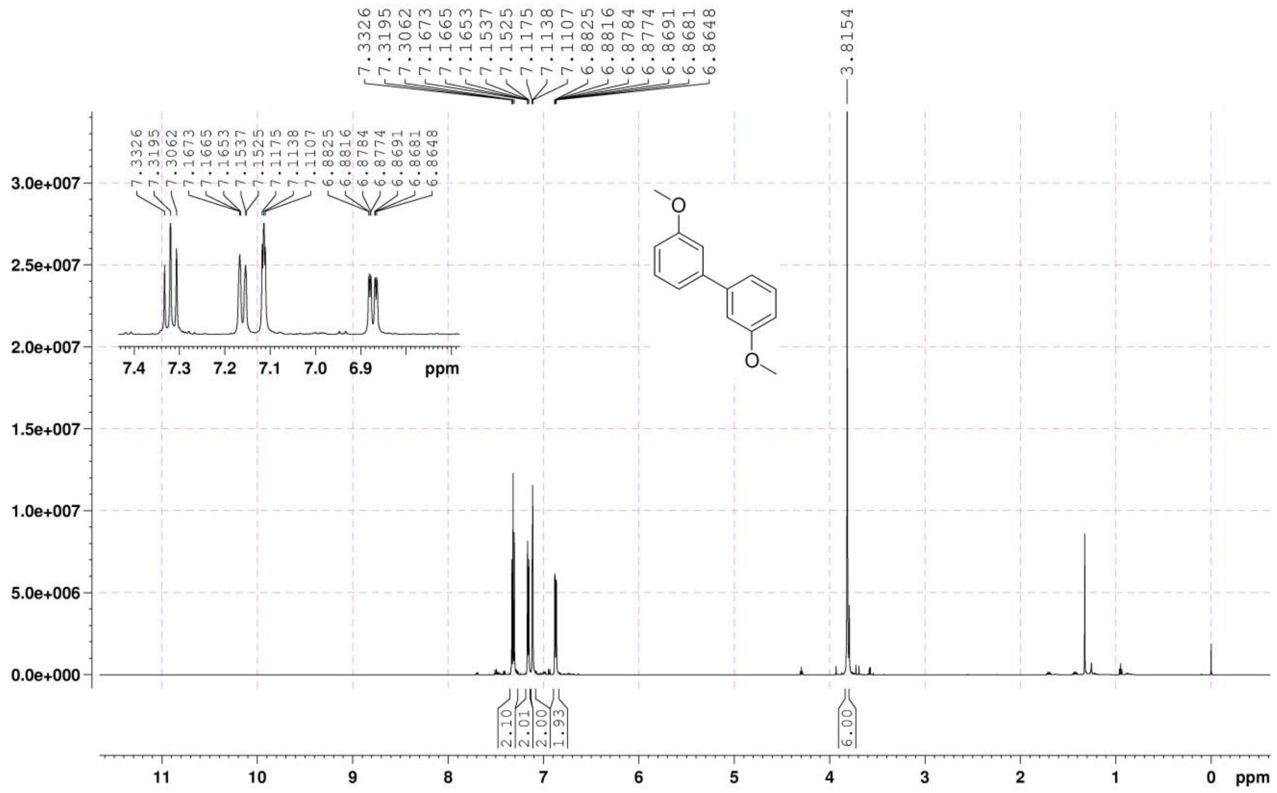

7.3326
7.3195
7.3062
7.1673
7.1665
7.1653
7.1537
7.1525
7.1175
7.1138
7.1107
6.8825
6.8816
6.8784
6.8774
6.8691
6.8681
6.8648
3.8154
2.10
2.01
2.00
1.93
6.00
ppm

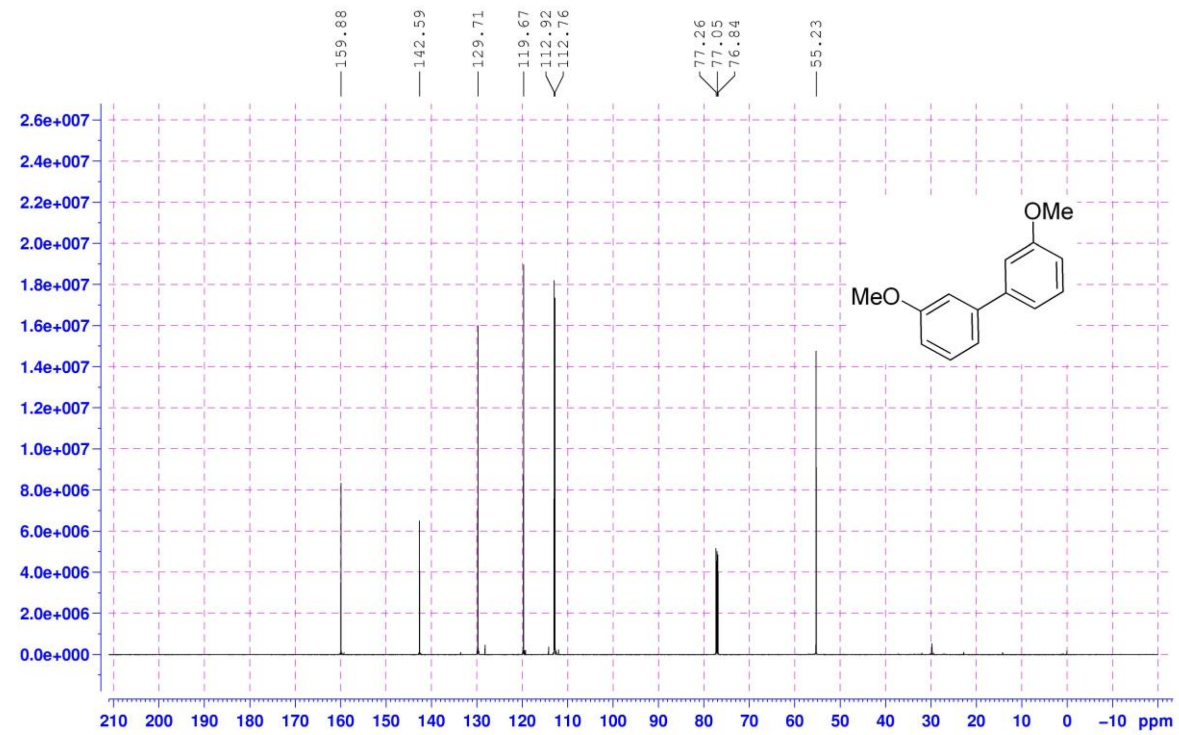

159.88
142.59
129.71
119.67
112.92
112.76
77.26
77.05
76.84
55.23
OMe
MeO
ppm

Compound 2e

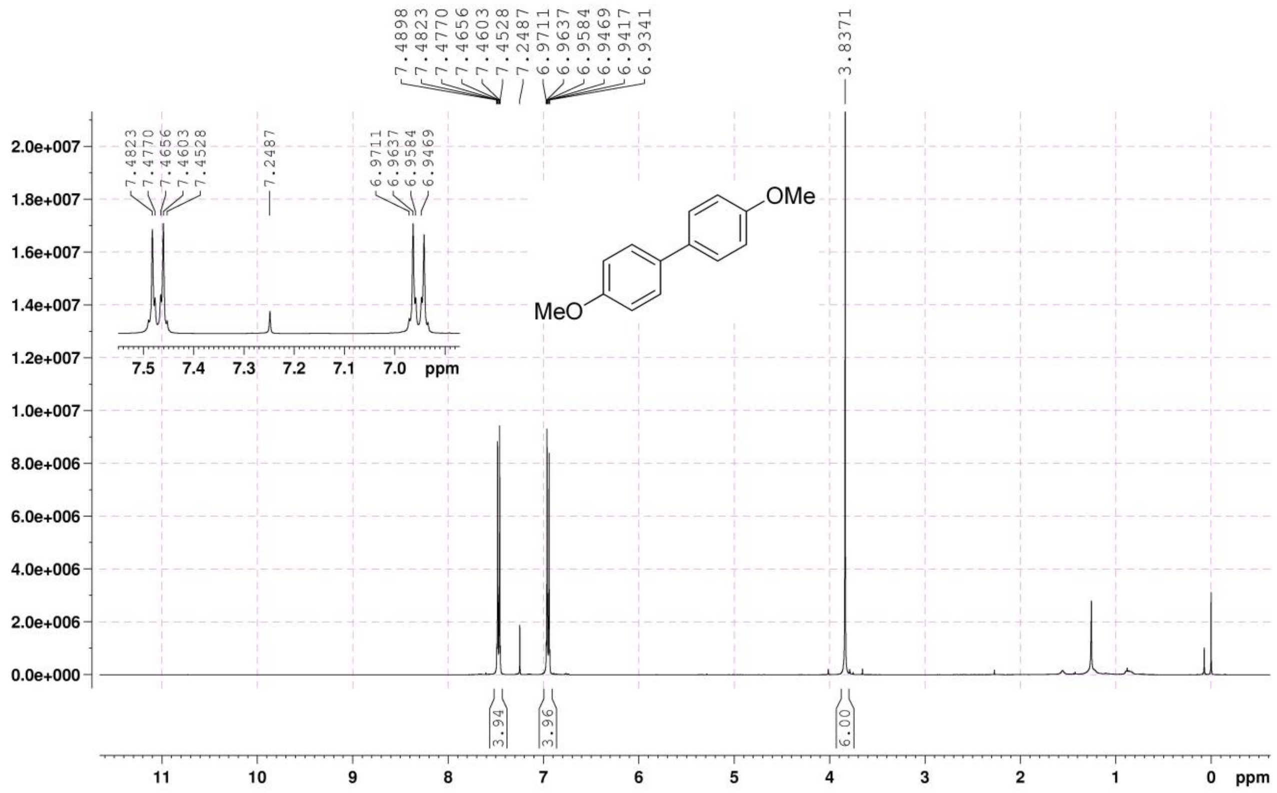
7.4898
7.4823
7.4770
7.4656
7.4603
7.4528
7.2487
6.9711
6.9637
6.9584
6.9469
6.9417
6.9341
3.8371
OMe
MeO
3.94
3.96
6.00
ppm

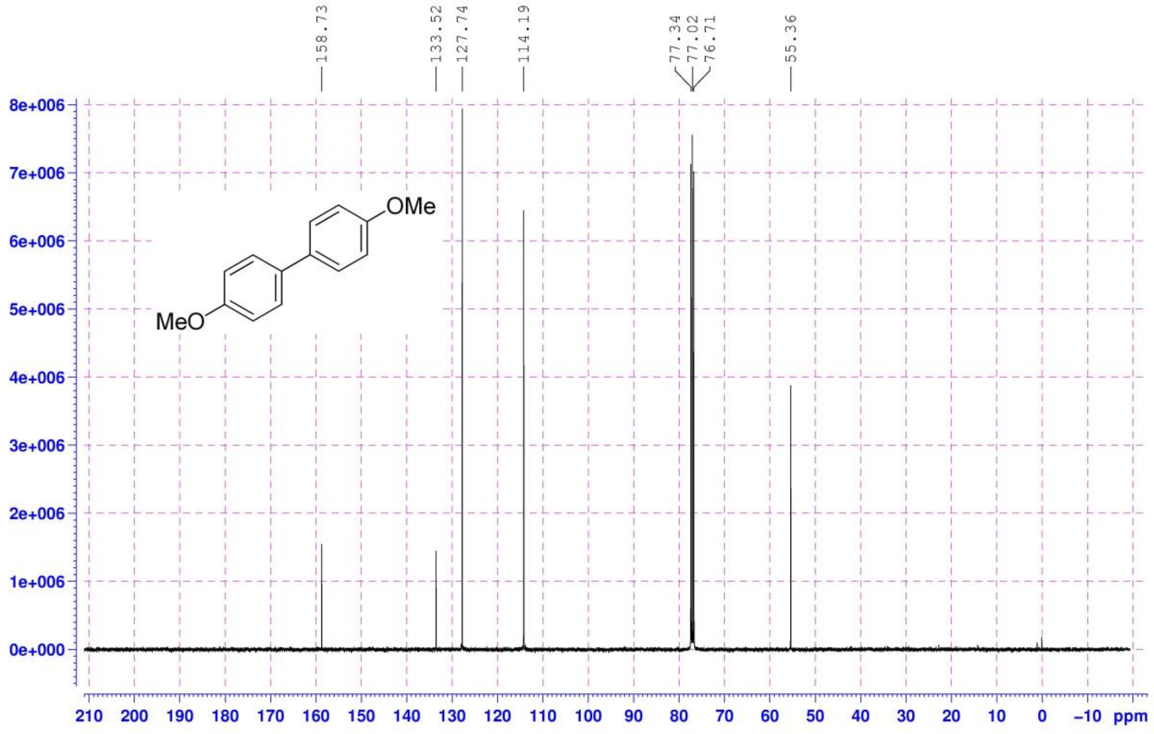
158.73
133.52
127.74
114.19
77.34
77.02
76.71
55.36
OMe
MeO
ppm

Compound 2f

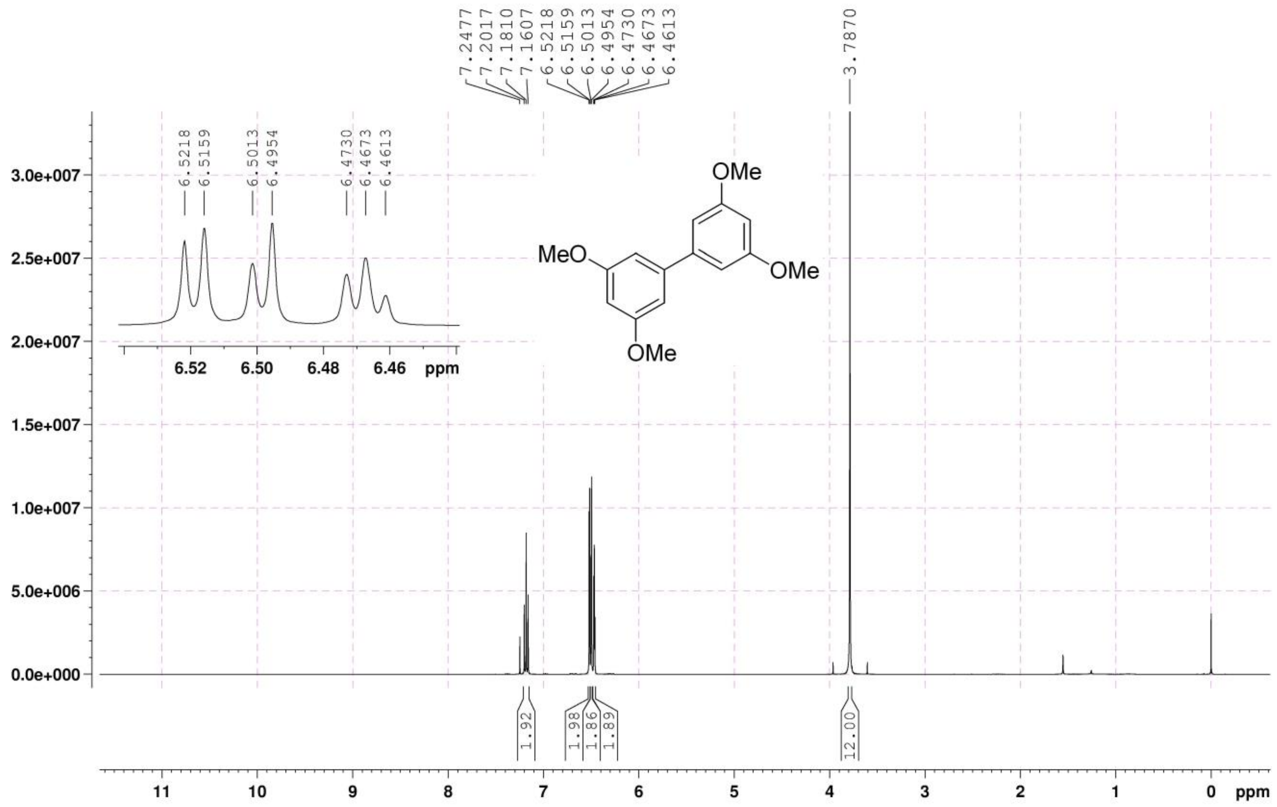
7.2477
7.2017
7.1810
7.1607
6.5218
6.5159
6.5013
6.4954
6.4730
6.4673
6.4613
3.7870
OMe
MeO
OMe
OMe
1.92
1.98
1.86
1.89
12.00
ppm

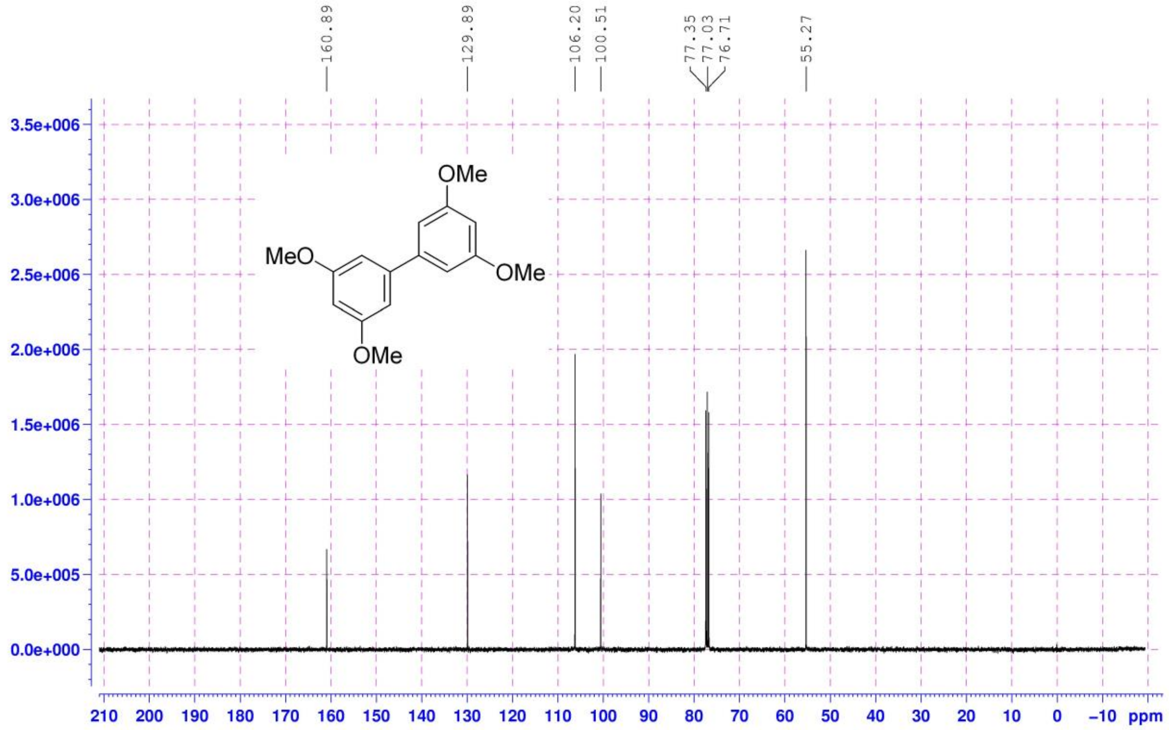
160.89
129.89
106.20
100.51
77.35
77.03
76.71
55.27
OMe
MeO
OMe
OMe
ppm

Compound 2g

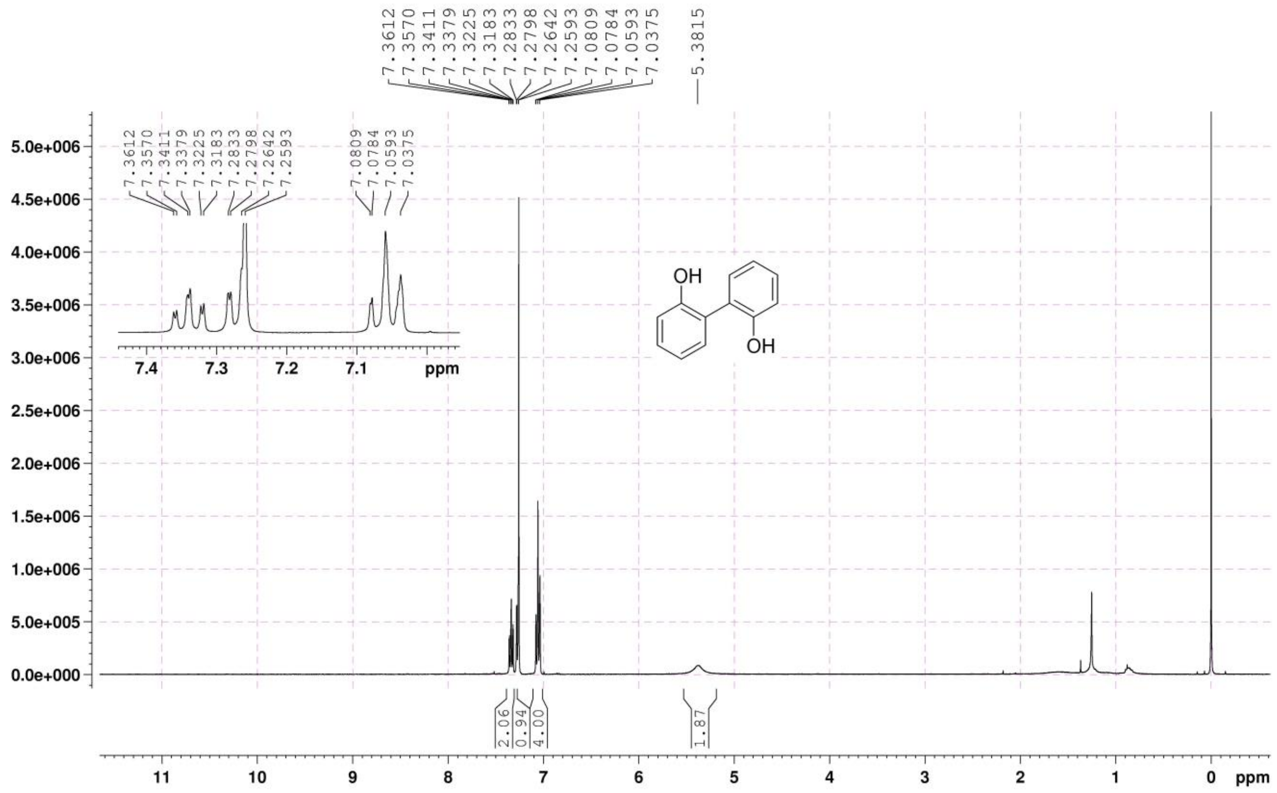
7.3612
7.3570
7.3411
7.3379
7.3225
7.3183
7.2833
7.2798
7.2642
7.2593
7.0809
7.0784
7.0593
7.0375
5.3815
OH
OH
2.06
0.94
4.00
1.87
ppm

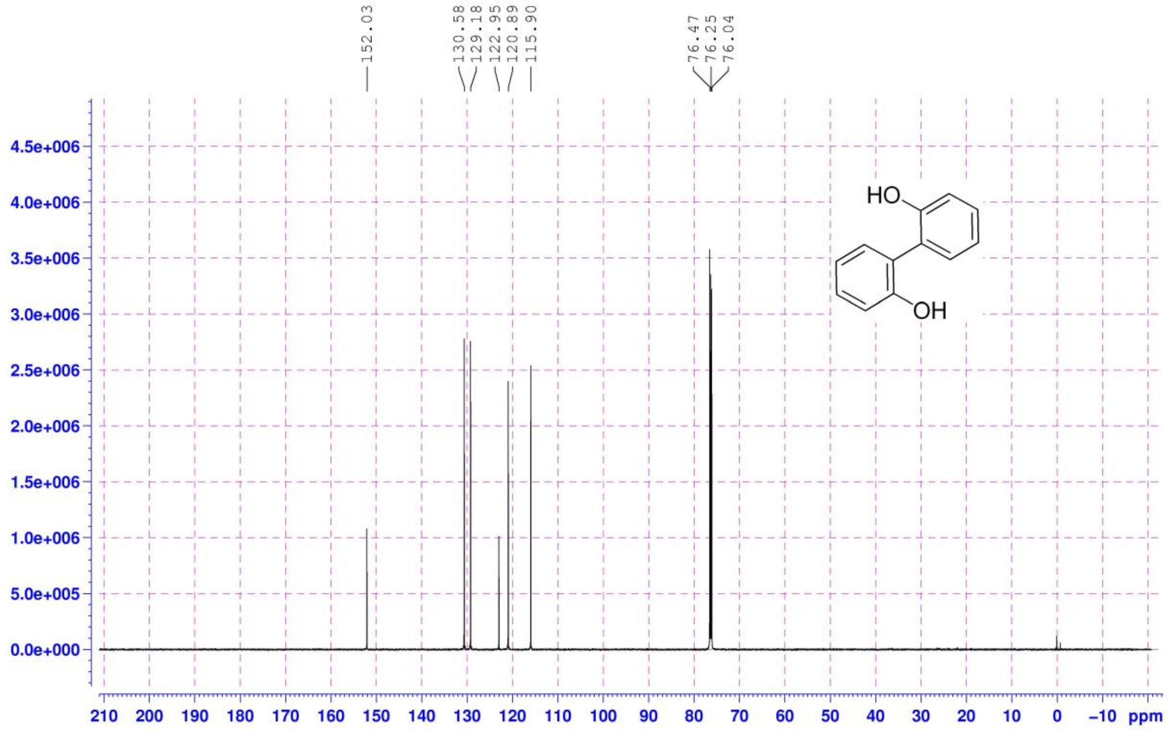
152.03
130.58
129.18
122.95
120.89
115.90
76.47
76.25
76.04
HO
OH
ppm

Compound 2h

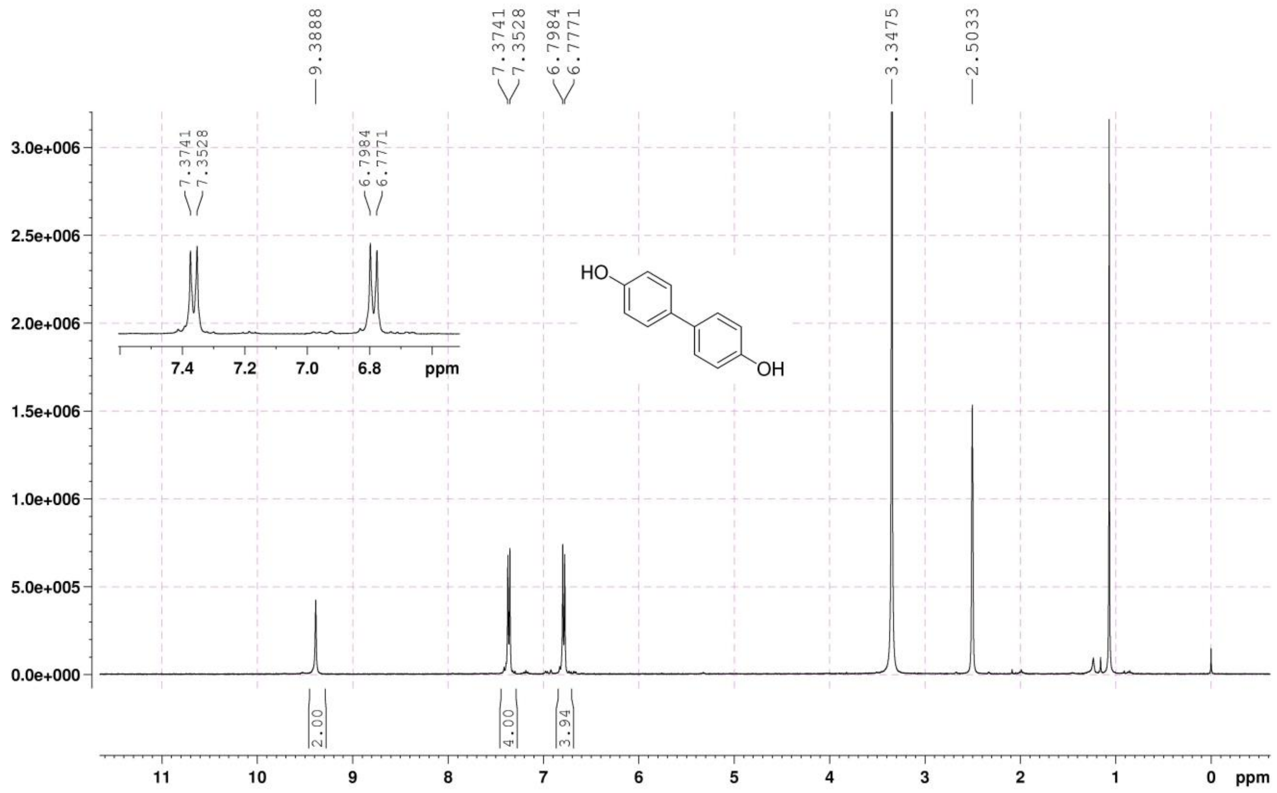
9.3888
7.3741
7.3528
6.7984
6.7771
3.3475
2.5033
HO
OH
2.00
4.00
3.94
ppm

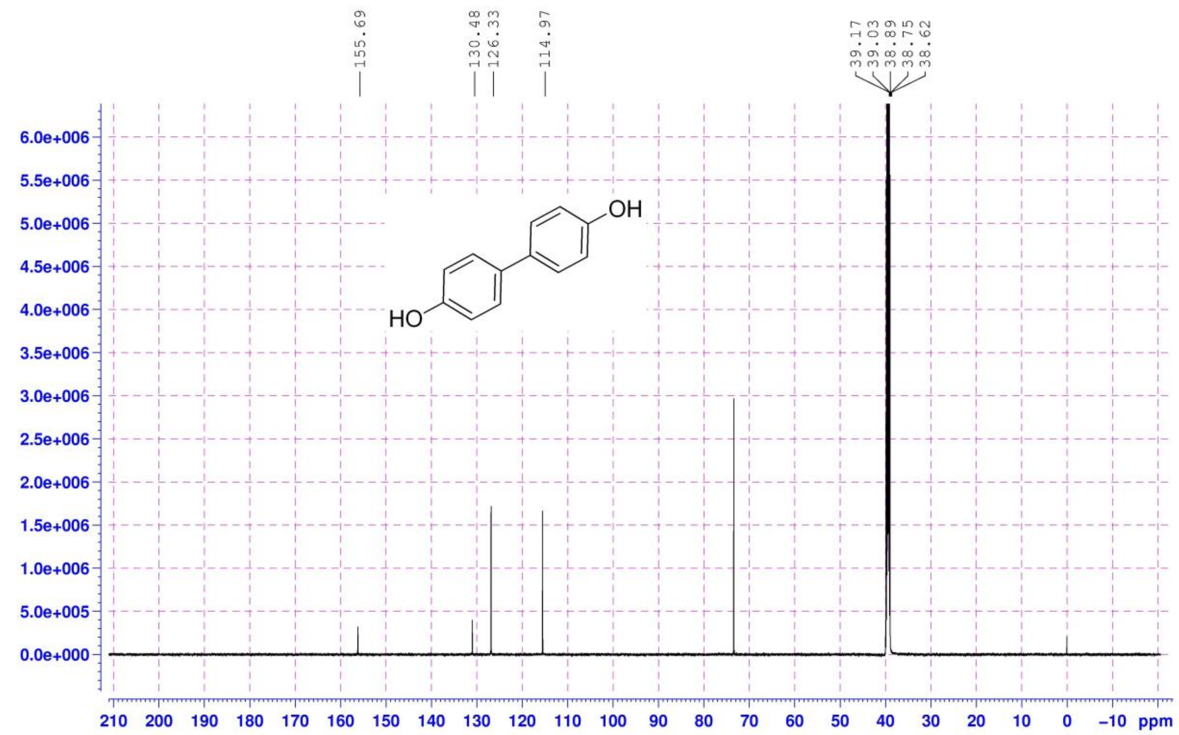
155.69
130.48
126.33
114.97
39.17
39.03
38.89
38.75
38.62
OH
HO
ppm

Compound 2i

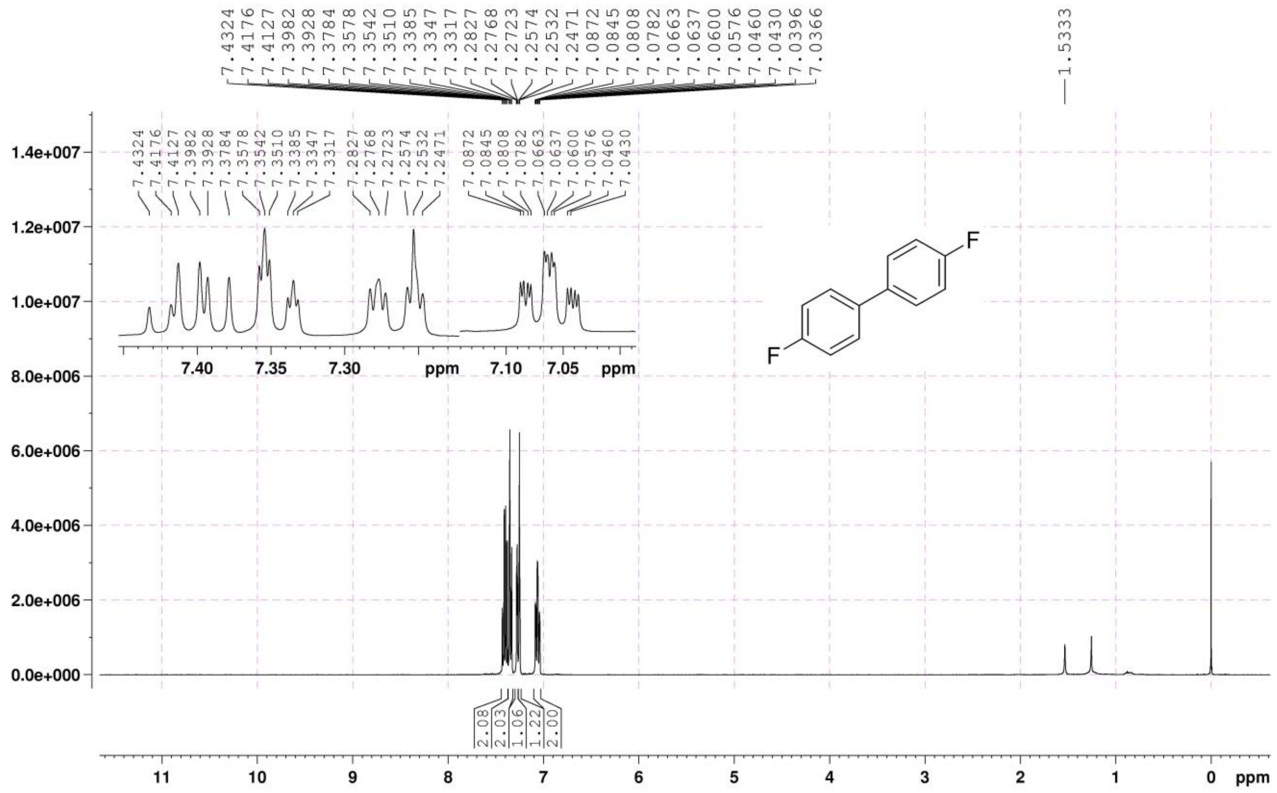

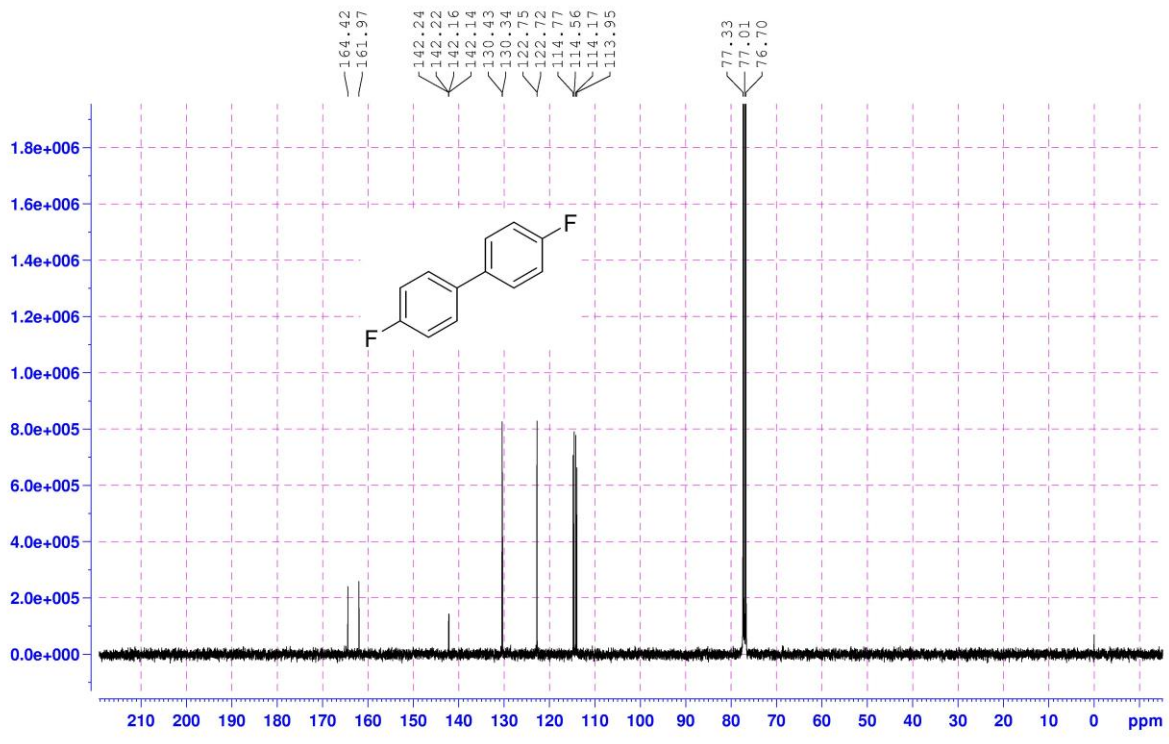

Compound 2j

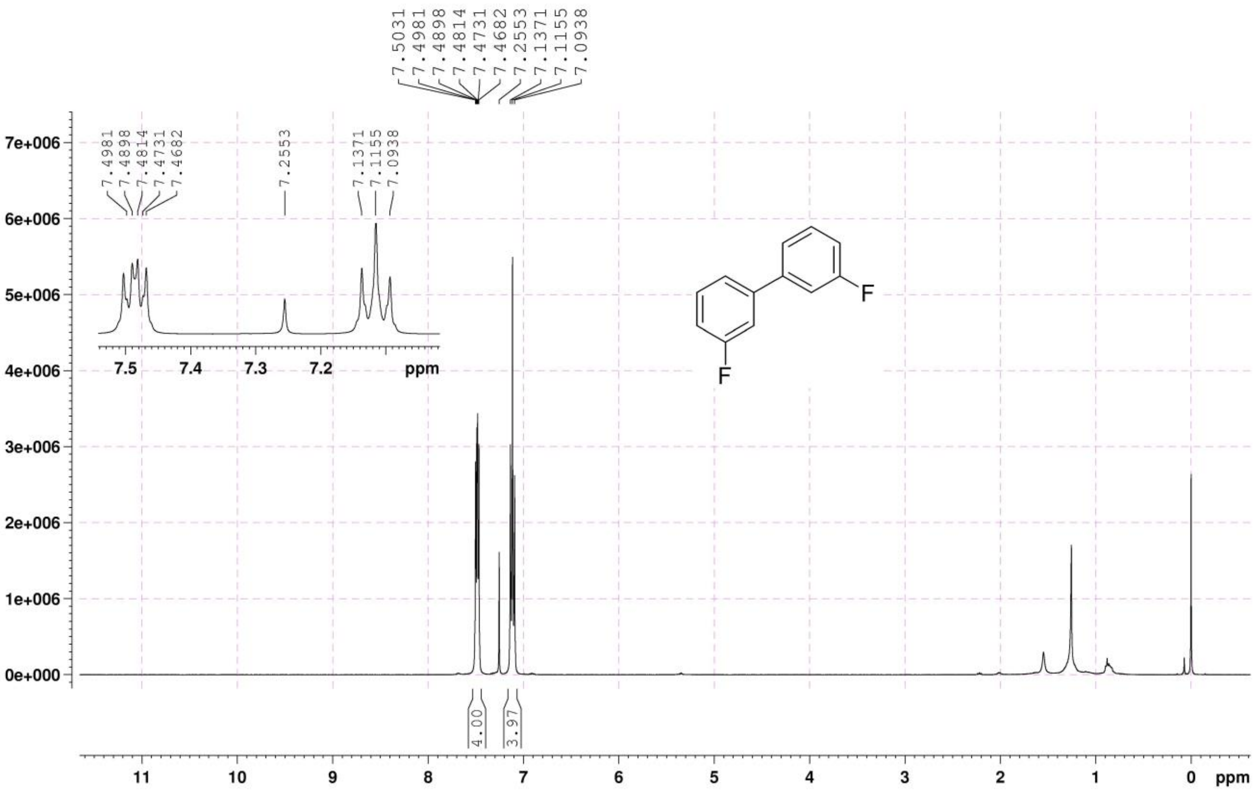
7.5031
7.4981
7.4898
7.4814
7.4731
7.4682
7.2553
7.1371
7.1155
7.0938
4.00
3.97
ppm
F
F

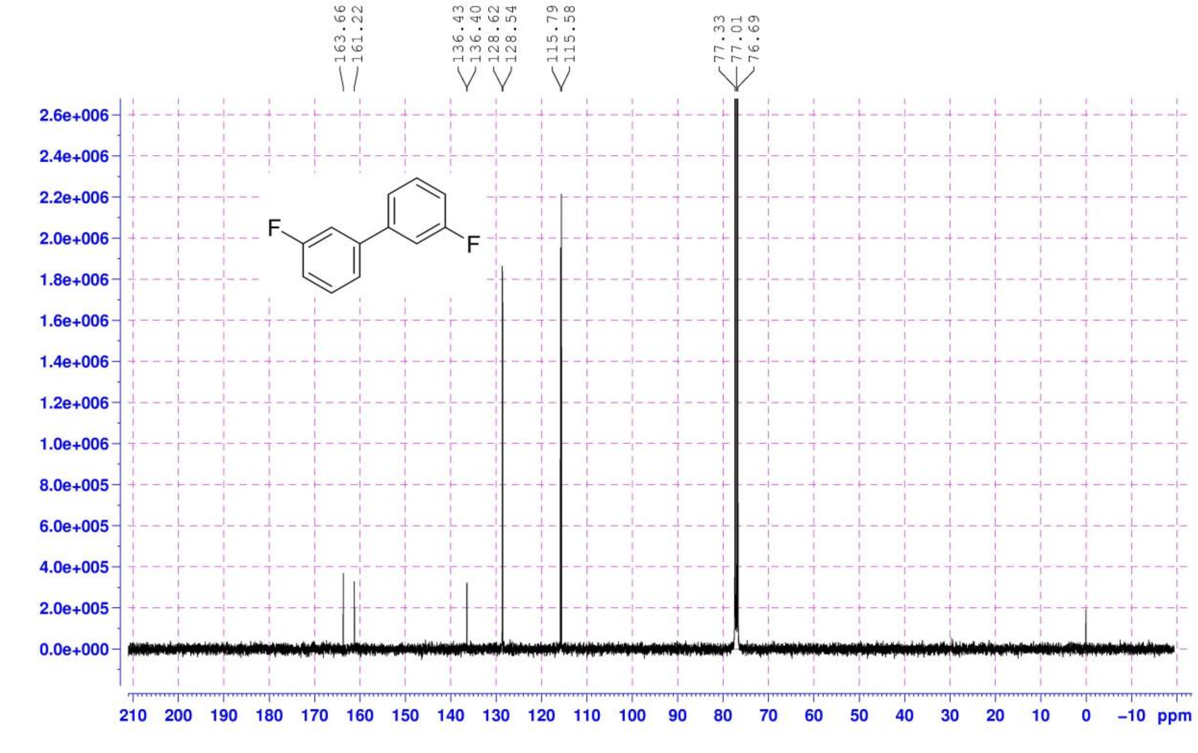
163.66
161.22
136.43
136.40
128.62
128.54
115.79
115.58
77.33
77.01
76.69
ppm
F
F

Compound 2k

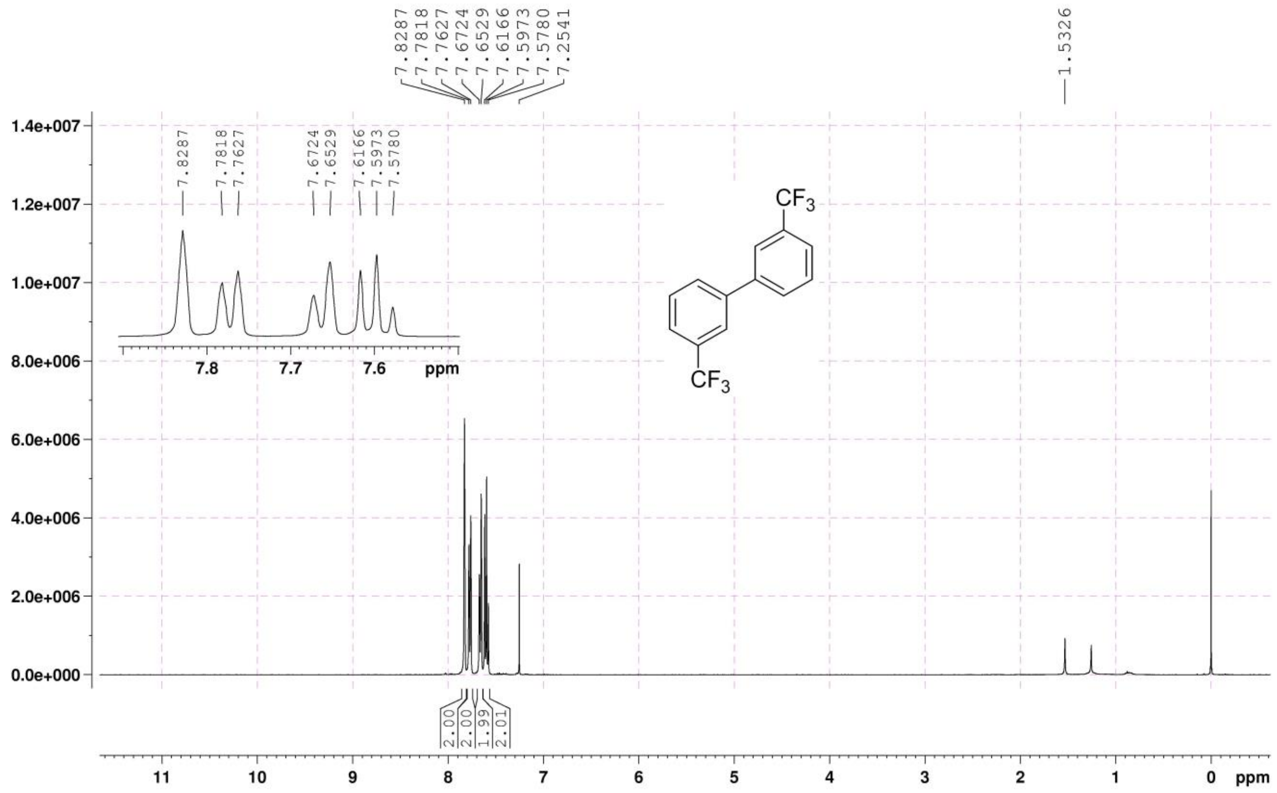
7.8287
7.7818
7.7627
7.6724
7.6529
7.6166
7.5973
7.5780
7.2541
1.5326
CF3
CF3
2.00
2.00
1.99
2.01
ppm

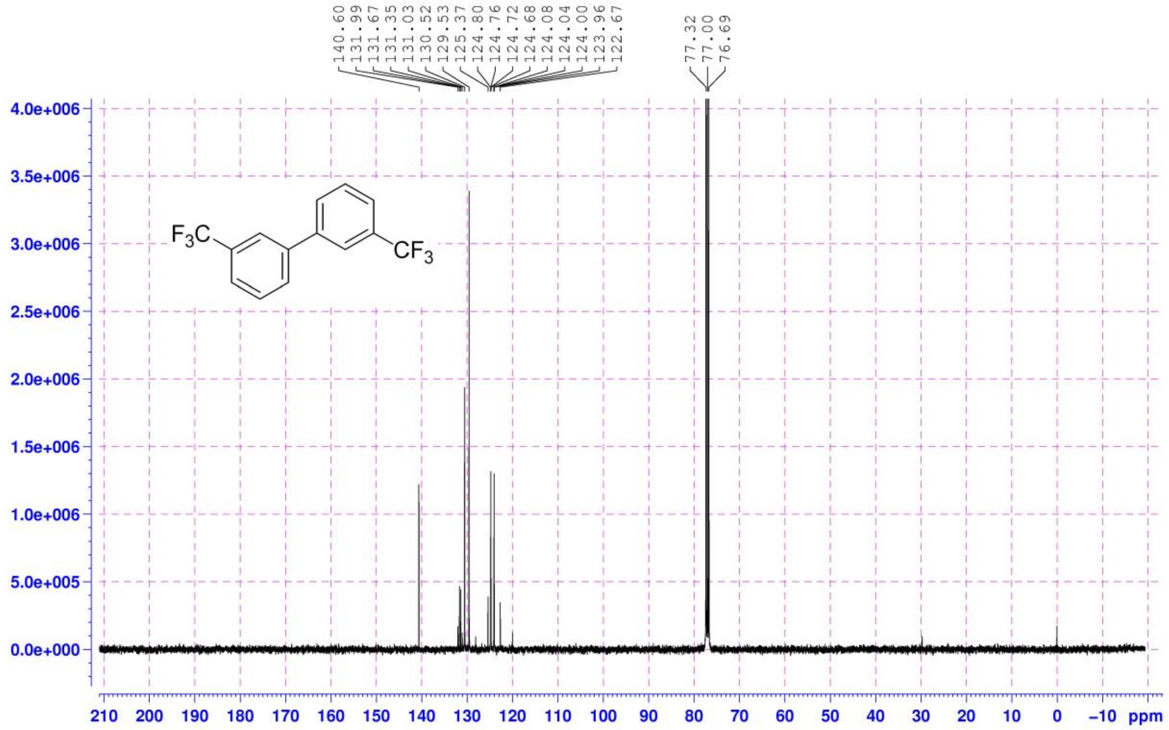
140.60
131.99
131.67
131.35
131.03
130.52
129.53
125.37
124.80
124.76
124.72
124.68
124.08
124.04
124.00
123.96
122.67
77.32
77.00
76.69
F3C
CF3
ppm

Compound 2l

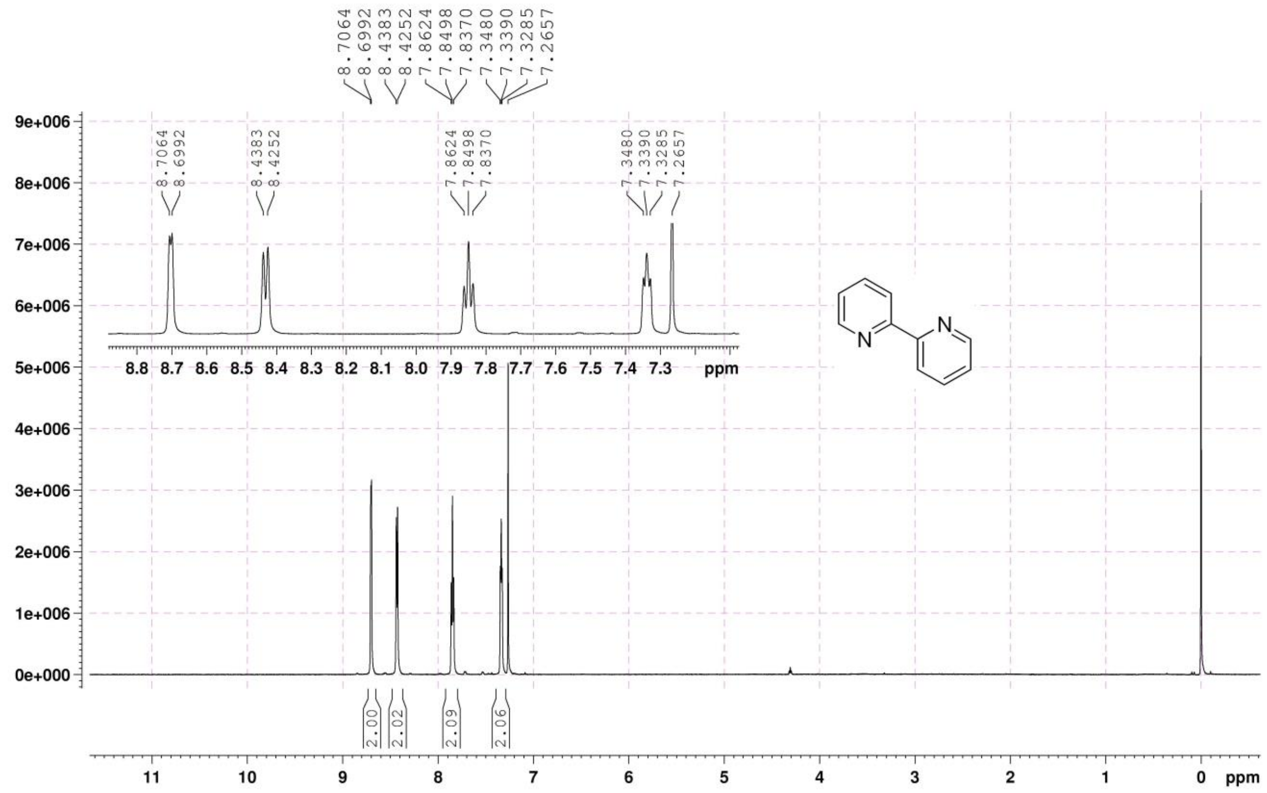
8.7064
8.6992
8.4383
8.4252
7.8624
7.8498
7.8370
7.3480
7.3390
7.3285
7.2657
2.00
2.02
2.09
2.06
ppm

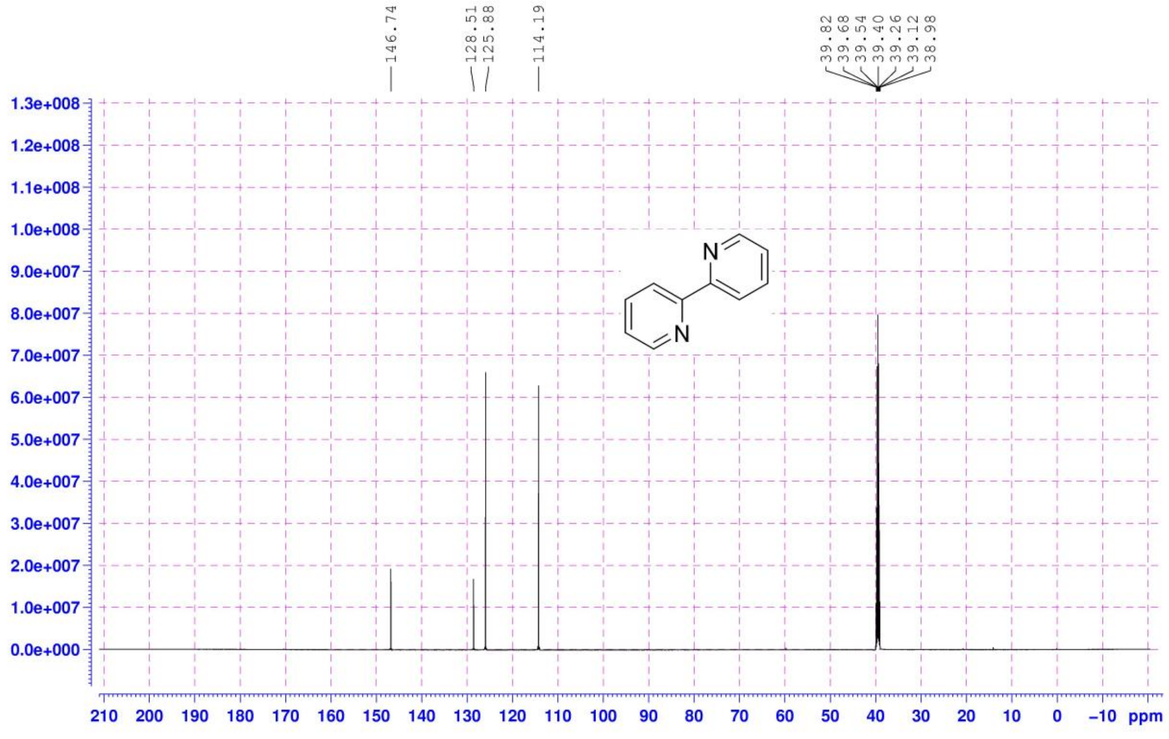
146.74
128.51
125.88
114.19
39.82
39.68
39.54
39.40
39.26
39.12
38.98
ppm

Compound 2m

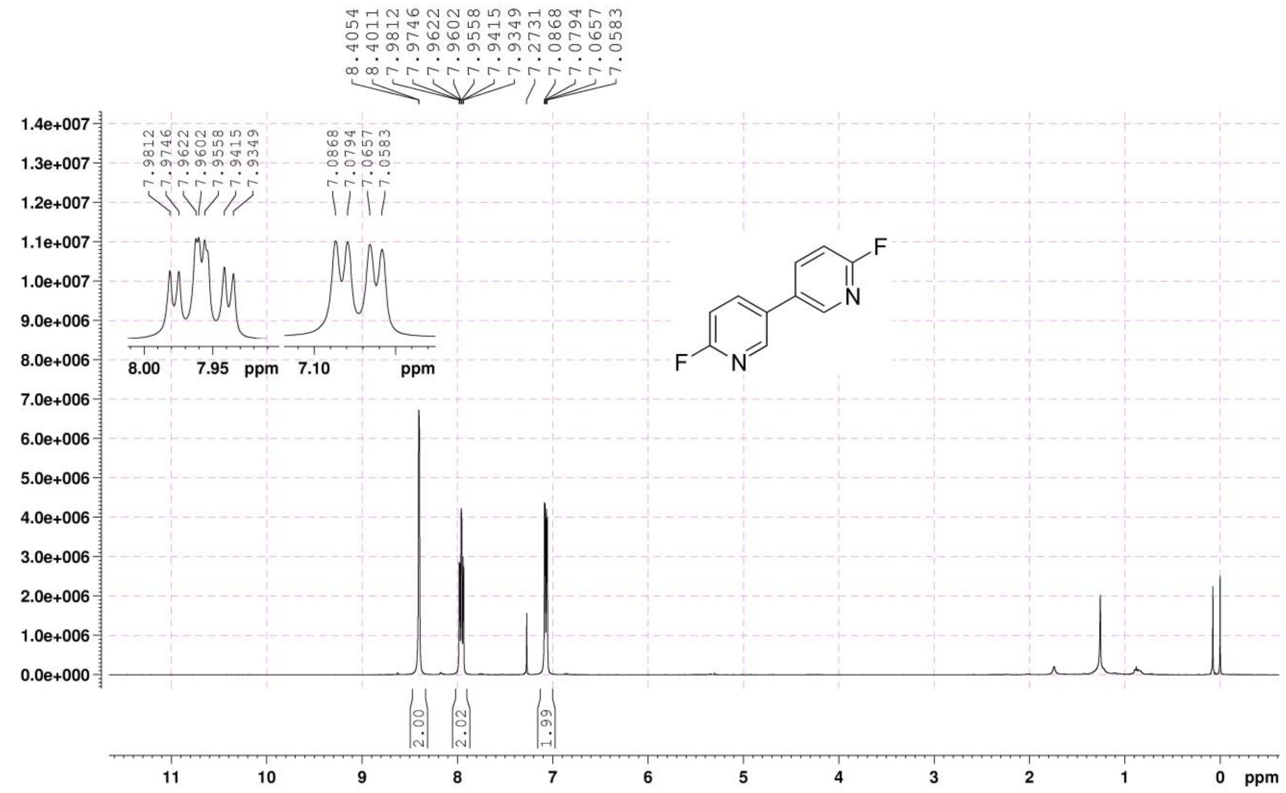
8.4054
8.4011
7.9812
7.9746
7.9622
7.9602
7.9558
7.9415
7.9349
7.2731
7.0868
7.0794
7.0657
7.0583
2.00
2.02
1.99
F
N
ppm

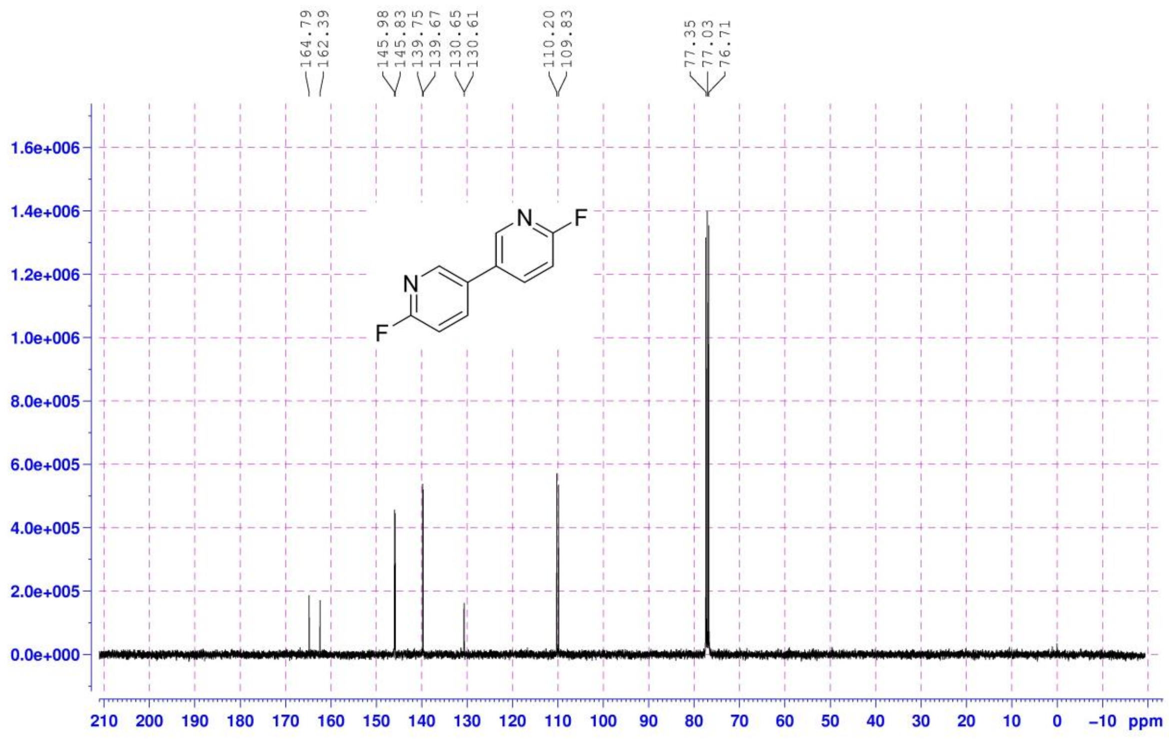
164.79
162.39
145.98
145.83
139.75
139.67
130.65
130.61
110.20
109.83
77.35
77.03
76.71
N
F
ppm

Compound 2n

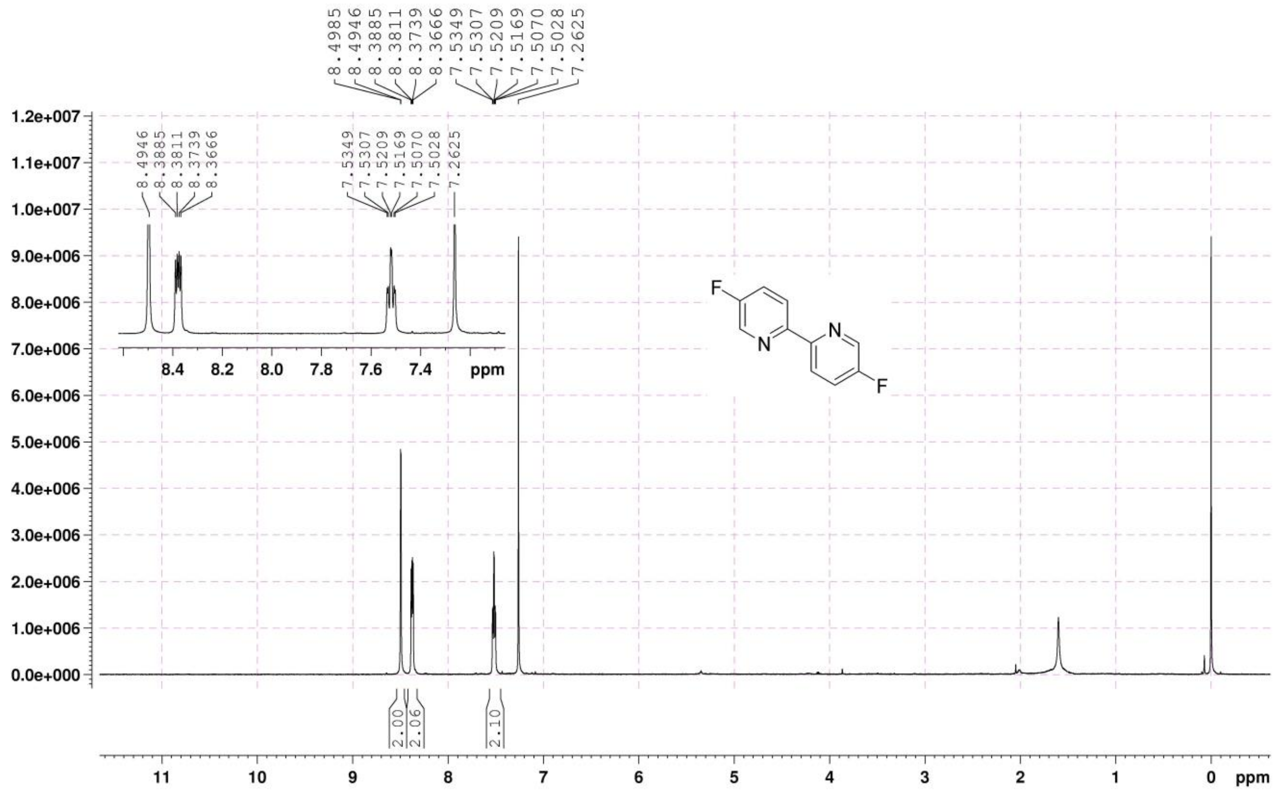
8.4985
8.4946
8.3885
8.3811
8.3739
8.3666
7.5349
7.5307
7.5209
7.5169
7.5070
7.5028
7.2625
2.00
2.06
2.10
ppm

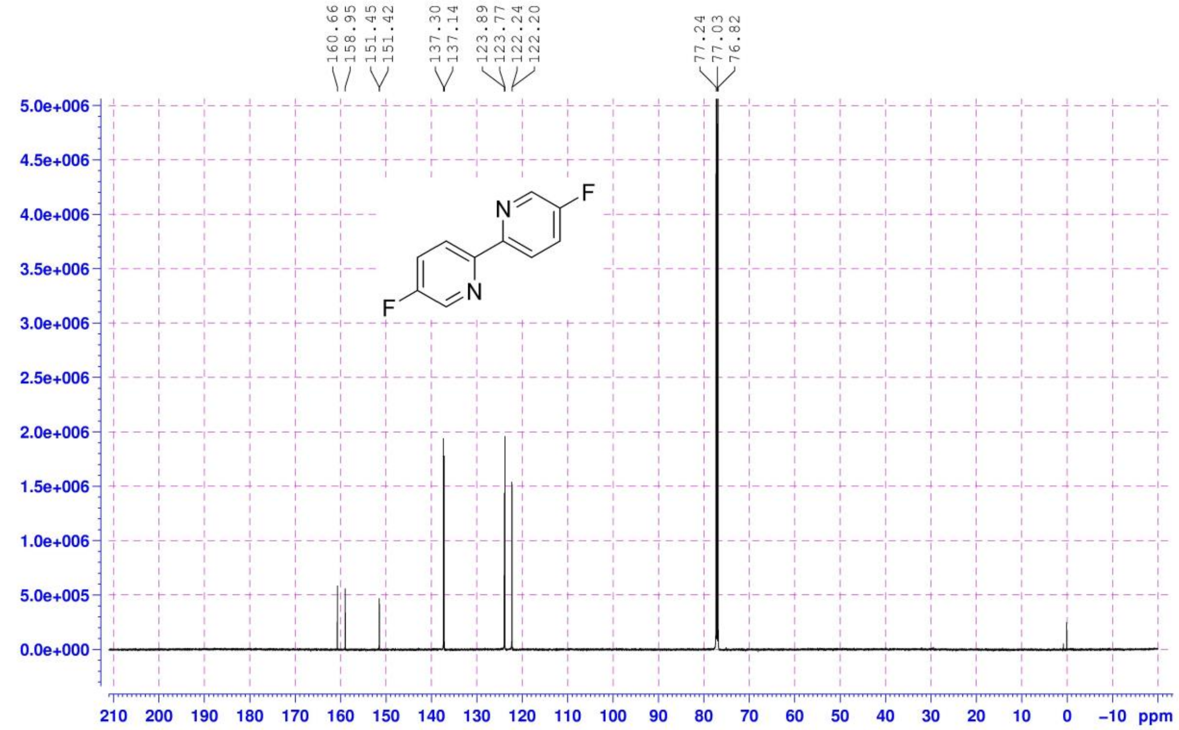
160.66
158.95
151.45
151.42
137.30
137.14
123.89
123.77
122.24
122.20
77.24
77.03
76.82
ppm

Compound 2o

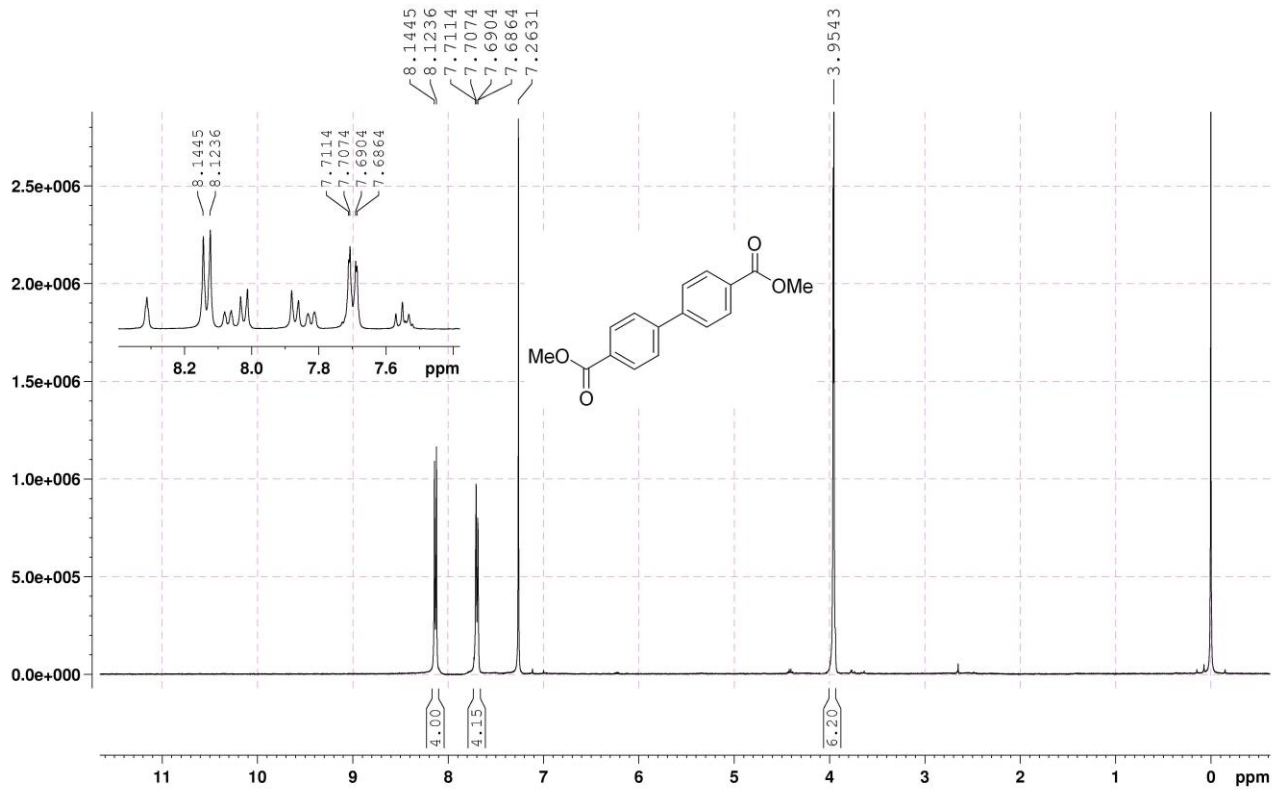
8.1445
8.1236
7.7114
7.7074
7.6904
7.6864
7.2631
3.9543
MeO
OMe
O
O
4.00
4.15
6.20
ppm

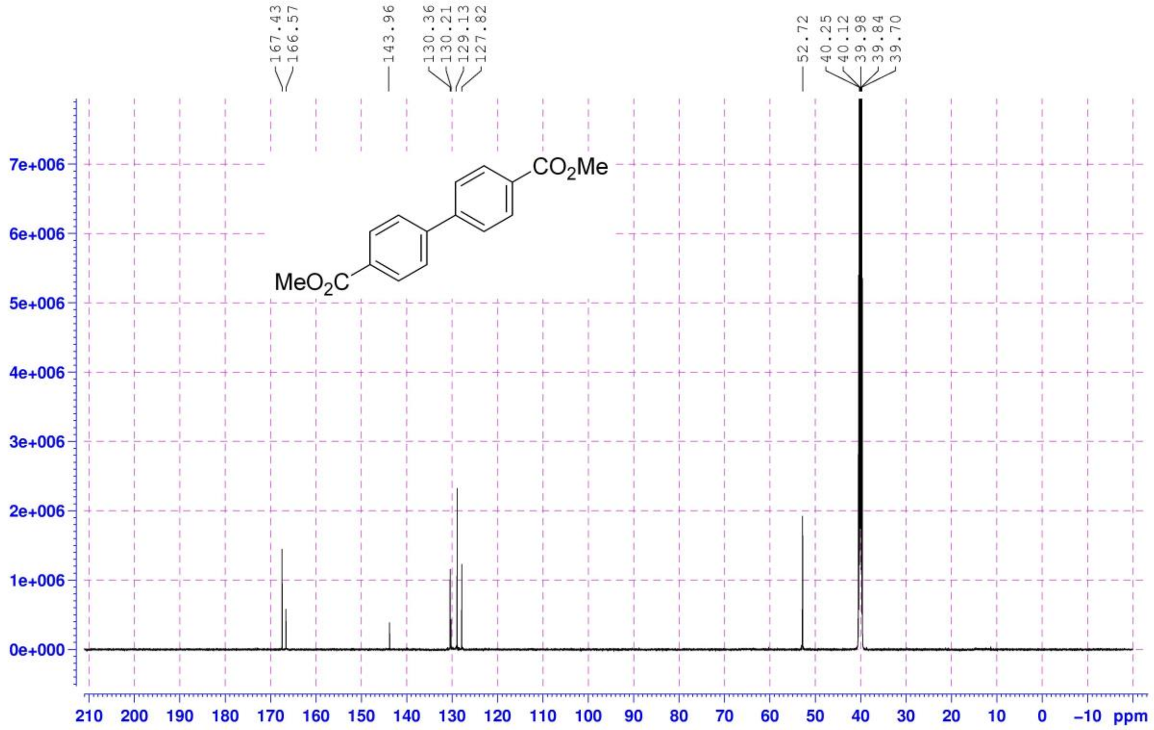
167.43
166.57
143.96
130.36
130.21
129.13
127.82
52.72
40.25
40.12
39.98
39.84
39.70
CO_2Me
MeO_2C
ppm

Compound 2p
